# Feasibility of glycated haemoglobin target-setting in adults with diabetes: A mixed-methods study

**DOI:** 10.1371/journal.pone.0317162

**Published:** 2026-01-05

**Authors:** Samuel J. Westall, Simon Watmough, Ram Prakash Narayanan, Greg Irving, Kevin John Hardy

**Affiliations:** 1 Faculty of Health, Social Care and Medicine, Edge Hill University, Ormskirk, United Kingdom; 2 Department of Diabetes and Endocrinology, Mersey and West Lancashire Teaching Hospitals NHS Trust, St Helens Hospital, Marshalls Cross Road, St Helens, United Kingdom; National Healthcare Group, SINGAPORE

## Abstract

**Background:**

HbA_1c_ targets guide diabetes management to reduce complications, yet their psychological effects are poorly understood. This feasibility study evaluated the practicality of conducting a definitive trial evaluating the impact of explicit HbA_1c_ target-setting in adults with diabetes.

**Methods:**

We conducted a randomised mixed-methods feasibility study. Adults with diabetes were allocated 1:1 to receive an explicit HbA_1c_ target set 5 mmol/mol above (Group A) or below (Group B) their current HbA_1c_. Biomedical (HbA_1c_, blood pressure, BMI) and psychometric patient-reported outcomes were measured at baseline and 3 months. Quantitative data were analysed in SPSS using independent-sample t-tests or Mann–Whitney U tests for between-group comparisons, and paired t-tests or Wilcoxon signed-rank tests for within-group changes. Qualitative data from semi-structured interviews with patients and healthcare professionals were analysed using the Framework Method of thematic analysis in NVivo. Acceptability was assessed via interview, and mixed-methods findings were integrated through triangulation to enhance validity.

**Results:**

Fifty participants were recruited; 34% withdrew. Though not powered to determine statistical significance, no between-group differences were observed in HbA_1c_ or patient-reported outcomes. Across groups, diabetes distress decreased, self-efficacy improved, and HbA_1c_ improved. Interviews indicated high acceptability and identified key motivators (target achievability, hypoglycaemia avoidance) and demotivators (limited understanding, perceived unattainability).

**Discussion:**

A randomised mixed-methods approach to HbA_1c_ target-setting is feasible and acceptable, providing methodological insights for a definitive trial.

**Trial registration:**

The study is registered with the ISRCTN (registration number: 12461724; date registered: 11^th^ June 2021).

## Introduction

### Background

Glycated haemoglobin (HbA_1c_) is central to guiding diabetes management and predicting future risk of complications. Contemporary guidelines emphasise individualising HbA_1c_ targets according to patient characteristics such as age, comorbidity, frailty, and risk of hypoglycaemia [1–12]. This personalised approach is endorsed in the joint ADA/EASD consensus statement, reflecting the growing focus on patient-centred care [13,14].

Sub-optimal HbA_1c_ control remains a major contributor to diabetes complications. Persistently elevated levels increase the risk of micro- and macro-vascular disease, leading to blindness, renal failure, cardiovascular events, stroke, amputation, and higher rates of dementia and cancer [15–21]. Despite pharmacological advances, only around 30% of people with Type 1 diabetes and 70% with Type 2 diabetes in England and Wales meet recommended HbA_1c_ targets [22], with similar patterns reported globally [23].

Multiple factors hinder achievement of optimal HbA_1c_ levels. Diabetes is strongly associated with depression, distress, and reduced self-efficacy, which negatively affect self-care, treatment adherence, and quality of life [3,4,24–26]. From a patient perspective, low motivation, limited understanding, and mental health comorbidity can impede glycaemic control [23]. From a healthcare perspective, complex management options and variable guideline recommendations challenge consistent target-setting [27]. While patient education has improved awareness of treatment goals [28], gaps persist in both knowledge and access to evidence-based support [23,29,30].

Clinicians increasingly recognise the benefits of individualised HbA_1c_ targets for reducing diabetes complications [5,31–34]. However, little evidence exists on how the process of setting explicit targets—particularly when initiated by clinicians—affects patients’ psychological well-being and self-management. Understanding the acceptability and preliminary impact of HbA_1c_ target-setting could inform more effective and psychologically attuned diabetes care.

## Aims and objectives

### Aims

The aim of this study was to evaluate the preliminary impact of setting explicit HbA_1c_ targets—either higher or lower than current levels—on patient-reported outcomes (PROs) and HbA_1c_, while assessing the feasibility of conducting a larger trial.

### Objectives

The objectives of this study were to:

1. Evaluate feasibility through recruitment, retention, and adequacy of data collection.
2. Examine the preliminary impact of the intervention on:

◦ **Psychosocial outcomes**: health-related quality of life, diabetes distress, self-care, well-being, and diabetes-related self-efficacy.
◦ **Clinical outcomes**: HbA1c, blood pressure, and body mass index (BMI) at 3-month follow-up

1. Determine the acceptability of the intervention and study procedures using interview data.
2. Explore participant and healthcare professional perspectives on the intervention, study processes, and perceived impact on well-being.

## Materials and methods

The research protocol was prospectively registered with the ISRCTN and available to view online (ISRCTN 12461724). A detailed description of the methods has been published and is available to view [35].

Briefly, the study was completed between May 2021 and May 2022. Setup and delivery was sponsored by St Helens and Knowsley Teaching Hospitals NHS Trust (ref: STHK-2021–003). This non-commercial single-site study was undertaken as part of the corresponding author’s PhD [36] in a salaried clinical research fellow post on-site at the NHS Trust. The study received ethical approval from the UK Health Research Authority (Cornwall and Plymouth Research Ethics Committee, REC reference 21/SW/0043, IRAS ID: 291254, 30^th^ April 2021).

### Participants

Eligible participants were adults aged 18 years and over with a diagnosis of type 1 or type 2 diabetes and a glycated haemoglobin (HbA1c) level between 64 and 125 mmol/mol at recruitment. Participants were identified through a local secondary care adult diabetes clinic. Individuals were excluded if they had a history of cardiovascular disease events, experienced severe hypoglycaemia within the past 12 months, or had hypoglycaemia unawareness (defined as a Gold score ≥ 4). Additional exclusions included unwillingness to self-monitor blood glucose or inject insulin if clinically required, a body mass index ≥ 45 kg/m², current participation in another clinical trial, pregnancy, requirement for regular venesection or blood transfusion, use of medical therapies known to affect glycaemic control (e.g., corticosteroids), serious illness likely to limit survival, or any factors likely to impair adherence to study procedures. Individuals who had opted out of research contact under the NHS national data opt-out service were also excluded. Eligible individuals were invited to participate, and reasons for declining were recorded.

### Recruitment

**Fig 1:**
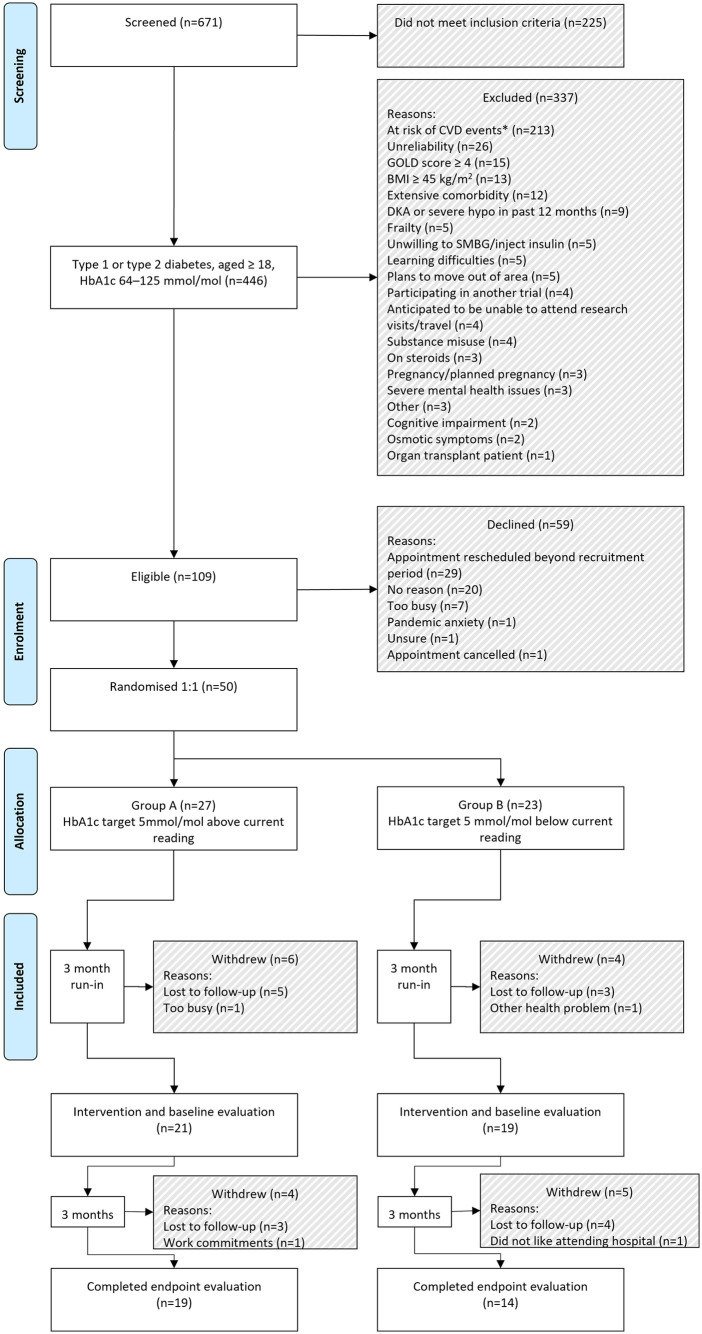
CONSORT flow diagram showing reasons for exclusion, dissent and withdrawal.

We planned to recruit a minimum of 50 patients attending secondary care diabetes clinic appointments over a 4-month period. Fig 1 details the number screened, eligible and randomised, with reasons for exclusion or withdrawal from the study. Sample size was determined according to the published protocol [35]. Recruitment was undertaken by the researcher working in liaison with the diabetes team to identify individuals who met the inclusion and exclusion criteria. An invitation letter with accompanying participant information sheet was posted to eligible individuals prior to their clinic visit. All patient-facing study-related documents were reviewed for face and content validity with a service user group consisting of a range of people from the general public, including people with diabetes and expert patients. Feedback was received via email and video call from ten individuals. Revisions to documents included clarity of language and the addition of researcher contact details on study documents. Written informed consent was obtained from all participants, with the original copy filed in the study site files and copies of signed documentation provided to participants.

A stratified purposive sample of participants involved in the study were selected for semi-structured interviews with the researcher, with an estimated 15 participants required. Sampling continued until the information power of the sample was deemed satisfactory by agreement between members of the research team (SJW, SW and GI). Additionally, a convenience sample of diabetes healthcare professionals were interviewed to obtain an additional perspective on the use of glycated haemoglobin targets in people with diabetes.

### Intervention

Participants were randomly allocated (stratified – Type 1 Diabetes/Type 2 Diabetes, random permuted block 1:1, as per protocol [35]) to receive relaxed or intensified glycated haemoglobin targets as described using the TIDieR checklist in Table 1 [37], extracted from the published protocol [35].

**Table 1:** Study intervention described using the TIDieR checklist [37].

Item	Description
1. Name of intervention	Group A: In-person (1-on-1) glycated haemoglobin target set to 5 mmol/mol above current participant glycated haemoglobin reading. Group B: In-person (1-on-1) glycated haemoglobin target set to 5 mmol/mol below current participant glycated haemoglobin reading.
2. Rationale	The psychological effect of glycaemic target-setting in people with diabetes is unknown.
3. Materials	A supplemental study leaflet shows a visual representation of participant’s current glycated haemoglobin result (on a scale from ‘*non-diabetic*’ to ‘*very poor glycaemic control*’) alongside participants’ study target glycated haemoglobin.
4. Procedures	The intervention took place as a discussion between the intervention provider and the participant. The discussion was guided by pre-defined script.
5. Intervention provider	The intervention was provided by the Main Investigator.
6. Mode of intervention delivery	The intervention was delivered face-to-face on an individual basis.
7. Location	The intervention was delivered in a private consultation room within a secondary care diabetes centre in the Northwest of England.
8. Time requirements/timing	The intervention took place 3 months post-allocation to allow for initial secondary care clinical management (undertaken at study entry) to be fully established. Intervention delivery took 10 − 15 minutes as a discussion between researcher and participant.
9. Personalisation	Not applicable.
10. Modifications	None.
11. Ensuring intervention adherence	A single intervention provider delivered the intervention to ensure rigorous and consistent adherence to the protocol for the duration of the trial.
12. Intervention delivery	A total of 40 participants (80%) in main study A received the intervention as described, with similar proportions between randomisation groups.

A schedule of procedures and events is presented in Table 2, in accordance with the published protocol and the 2013 SPIRIT statement [35,38].

**Table 2:** Schedule of procedures and assessments.

Study period	Pre-baseline	Baseline	Intervention	Endpoint	Interviews
Time point	–	0 months	3 months	6 months	0–12 months
**Screening**					
**Screen electronic records**	X				
**Postal invitation**	X				
**Enrolment**					
**Check eligibility**		X			
**Informed consent**		X			
**Allocation**		X			
**3-month run-in**		X	X		
**Intervention**					
**Group A or B intervention**			X		
**Assessments**					
**POC glycated haemoglobin**		X	X	X	
**BP, BMI**		X	X	X	
**EQ-5D-5L, PAID, SDSCA, W-BQ12, DES-LF**			X	X	
**Acceptability survey**				X	
**Additional elements**					
**Interview with participants**^**†**^					X
**Interview with HCPs**^**‡**^					X

**POC: point-of-care; HbA**_**1c**_ **: glycated haemoglobin; BP: blood pressure; BMI: body mass index; EQ-5D-5L: EuroQoL-5D-5L; PAID: Problem Areas in Diabetes; SDSCA: Summary of Diabetes Self-care Activities; W-BQ12: Well-being Questionnaire-12; DES-LF: Diabetes Empowerment Scale-Long Form; HCP: Healthcare Professional; mmol/mol: millimoles per mole, glycated haemoglobin SI unit of measurement**

† **Sub-study B. Semi-structured interviews in those enrolled in sub-study A.**

‡ **Sub-study C. Semi-structured interviews with healthcare professionals.**

§ **Sub-study D. In those declining to take part in sub-study A, survey and semi-structured interview.**

## Outcomes

### Quantitative feasibility outcome data

Rates of participant eligibility, recruitment, retention and questionnaire response, were captured and recorded in an anoymised database.

### Other quantitative data

Baseline and endpoint biomedical outcomes (HbA_1c_, BMI, BP) and PROs of health-related quality of life (Euro-QoL-5D-5L), diabetes-related distress (Problem Areas in Diabetes; PAID), self-care (Summary of Diabetes Self-Care Activities; SDSCA), well-being (Well-Being Questionnaire 12; W-BQ12), and self-efficacy (Diabetes Empowerment Scale-Long Form; DES-LF) were collected.

To contextualise the results, Euro-QoL-5D-5L outputs an index value with zero being an equivalent health state to death, anything less than zero equivalent to a health state worse than death, any positive number being a health state greater than death and 1.000 the maximum health state [39]. For PAID, scores greater than 40 indicate high distress and risk of emotional burnout, scores 17–39, moderate distress, and less than 17, low distress [40]. SDSCA measures the diabetes self-care activities of ‘general diet’, ‘specific diet’, ‘exercise’, ‘blood sugar testing’ and ‘foot care’ (scored on ‘number of good days per week’, 0 − 7) [41]. W-BQ12 measures wellbeing on a scale of zero to 36, with higher scores indicating better general wellbeing [42]. DES-LF measures diabetes-related psychosocial self-efficacy on a scale of one to five, with higher scores indicating higher levels of self-efficacy [43]. Our study protocol reports on the tool selection process, including data on internal consistency and responsiveness to change.

### Qualitative data

Two interview sub-studies were run alongside the quantitative study aspects. These sub-studies involved semi-structured interviews with feasibility study participants on study acceptability and their wellbeing, and interviews with healthcare professionals on the use of glycated haemoglobin targets in people with diabetes. Separate invitations letters and information sheets containing interview schedules were sent in advance to participants.

### Progression criteria

We pre-specified criteria to be met in recommending progression to a conclusive study. These criteria were: recruitment of 50 participants within the 4-month recruitment period, retention of 80% or more at endpoint and questionnaire response rate of 75% or more. Additionally, results of a Likert-type acceptability survey and qualitative data free-text and interview transcript data were considered when determining progression, including the acceptability of study processes.

### Data analysis

All data for each participant were recorded in anonymised form in a database. The completed dataset was imported into IBM statistical package for social sciences (SPSS) version 25 [44].

Descriptive statistics were used to present participant characteristics and primary feasibility outcomes. Baseline participant characteristics of interest were age, gender, diabetes type, diabetes duration, index of multiple deprivation decile, body mass index, blood pressure, medication usage and glycated haemoglobin.

Feasibility outcomes, presented as percentages, were rates of eligibility, recruitment, participant retention and per-item questionnaire response rate. Eligibility rate was calculated by dividing the total number of patients eligible for recruitment by the total number screened. Similarly, recruitment rate was calculated by dividing the number of participants recruited (eligible, agreed to take part, and signed consent) by the total number of eligible participants. Retention rate was determined at three- and six-months post recruitment by dividing the number of participants attending study visits by the total number of initial recruits. Reasons for non-attendance and/or withdrawal from the study were recorded. Response rate was determined by counting the number of non-blank responses for each item in each questionnaire and dividing by the total number of items. Response rate data were calculated for participants who completed the study and for all participants including those who did not attend. Following calculation of response rate, missing data in questionnaires were handled according to the accompanying questionnaire user guides. Additionally, descriptive statistics were used to summarise pre- and post-intervention glycaemic control (HbA_1c_) and patient-reported psychometric outcomes of health-related quality of life, self-care, diabetes-related distress, wellbeing, and psychosocial self-efficacy for completers.

Participant glycated haemoglobin levels (HbA_1c_) were monitored at recruitment and pre- and post-intervention using a point-of-care HbA_1c_ analyser maintained and calibrated as per NHS laboratory standards. Continuous variables from PROs and glycated haemoglobin values are presented as means (±standard deviation, SD and 95% confidence interval, CI) or medians (±interquartile range, IQR) depending on data skewness. Data skewness was determined using the Shapiro-Wilk test in SPSS alongside visual inspection of normal Q-Q plots and histograms. Where outliers were noted to skew data, they were removed prior to analysis. Inspection of histograms, normality plots and box plots allowed for identification of outliers and extreme outliers.

Outliers were defined as:

- any values less than [1st quartile – (1.5 x IQR)].
- any values greater than [3rd quartile + (1.5 x IQR)].

Extreme outliers were defined as:

- any values less than [1st quartile – (3 x IQR)].
- any values greater than [3rd quartile + (3 x IQR)].

Where outliers or extreme outliers were present and data distributions were skewed, outliers were removed, and data distributions re-evaluated.

Whilst recognising that feasibility studies are underpowered to consider the use of inferential statistics in determining statistically significant changes in pre- and post-intervention scores in psychometric outcomes, inferential statistics were used to understand any emerging trends in the data. We however recognise that the outcome measures presented here can give indications of the potential effectiveness of the intervention [45,46], though caution should be taken in interpreting these results. The difference between pre- and post-intervention scores (delta) is denoted by the Greek symbol, Δ. Where pre- and post-intervention psychometric outcome scores were continuous and normally distributed, the paired samples t-test was used. Where distributions were skewed, the Wilcoxon matched-pair signed-rank test was used. Differences between randomisation groups were determined using the independent-sample t-test or the Mann-Whitney U test where data were normally distributed or skewed, respectively.

Multiple linear regression analysis was conducted to identify confounding factors (gender, age, diabetes duration, IMD decile, BMI) potentially affecting outcomes.

Qualitative data from semi-structured interviews with patients and healthcare professionals were analysed using the Framework Method of thematic analysis [47] in NVivo qualitative data analysis support software [48]. Anonymised quotes from transcripts were coded for each pre-determined theme of interest with additional inductive themes added as they emerged. With emergent themes potentially arising in later transcripts, earlier transcripts were re-coded to encompass the additional themes. Coding strategy for transcript analysis was determined by discussion amongst the research team followed by consensus on a final coding strategy. Any disagreements were resolved with input from a senior reviewer (SW). This approach allowed contrast of themes from the patient and healthcare professional perspectives.

Interviewing participants taking part in the quantitative study aspects was undertaken to enable a deeper understanding of the themes of glycated haemoglobin target individualisation, mental health in diabetes and study acceptability and their relationship with health-related quality of life, self-care, diabetes-related distress, and wellbeing.

Source triangulation, as described by Lincoln and Guba [49], was enabled by contrasting qualitative data output from healthcare professionals with data from people with diabetes. This improved qualitative data credibility and consistency, enhancing the trustworthiness of findings. To check the consistency of the interview findings, method triangulation was used to compare the qualitative findings with quantitative results of health-related quality of life, self-care, diabetes-related distress, wellbeing, and diabetes-related psychosocial self-efficacy.

## Results

This study is reported using the CONSORT 2010 reporting guidelines for randomised control trials [50] (see supporting information, S1 File CONSORT Checklist). The CONSORT flow diagram is presented in Fig 1. There was no deviation from the approved study protocol (see supporting information S2 File Approved protocol). Feasibility data, PRO data, HbA_1c_ levels, BMI, BP, survey results and semi-structure interview transcripts comprise the results of this study. Participant characteristics are presented in Table 3. Participants were predominantly of white British ethnicity.

**Table 3:** Participant characteristics.

Variable	Group A – relaxed HbA_1c_ target (n = 27)	Group B – intensified HbA_1c_ target (n = 23)	P value
**Completers (%)**	70%	61%	.48
**Age (median [IQR])**	50 [14]	46 [36]	.54
**Gender (%female)**	30%	52%	.10
**T1DM (%)**	41%	39%	.91
**Recruitment BMI (mean [SD])**	31.1 [6.8]	31.6 [6.8]	.80
**Diabetes duration (mean [SD])**	9.9 [6.5]	10.4 [6.6]	.80
**IMD decile (median [IQR])**	3 [5]	2 [5]	.73
**Recruitment HbA**_**1c**_ **(mean [SD])**	83.1 [14.5]	85.9 [14.6]	.50
	**Completers (n = 33)**	**Non-completers (n = 17)**	**P value**
**Group A (%)**	58%	47%	.48
**Age (median [IQR])**	51 (17)	42 (26)	.04*
**Gender (%female)**	45%	29%	.27
**T1DM (%)**	33%	53%	.18
**Recruitment BMI (mean [SD])**	30.7 (6.7)	32.5 (6.7)	.38
**Diabetes duration (mean [SD])**	9.8 (6.0)	10.9 (7.5)	.59
**IMD decile (median [IQR])**	3 (5)	2 (5)	.90
**Recruitment HbA**_**1c**_ **(mean [SD])**	83.6 (12.8)	85.9 (17.5)	.60

**IMD = Index of Multiple Deprivation; IQR = interquartile range; SD = standard deviation**

**Fig 2:**
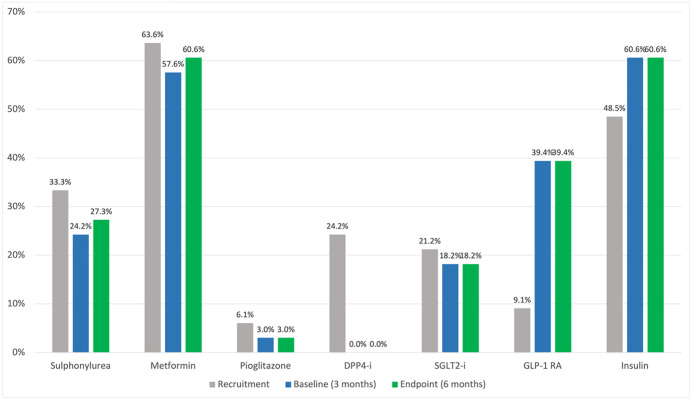
Bar chart showing medication usage at recruitment, baseline and endpoint.

Changes in medication during the run-in period are demonstrated in Fig 2. Predominant changes were cessation of DPP4-i class medications and commencement of insulins and GLP-1 RA class medications. Adjustments to participant diabetes medications between baseline and endpoint were minimal. Fig 2 demonstrates percentage medication usage by medication class at recruitment (0 months), baseline (3 months) and endpoint (6 months).

### Feasibility

We planned to recruit 50 participants into the quantitative aspects of the study. Electronic records of 671 people with diabetes due to attend secondary care diabetes clinics were screened for entry. Of those screened, 446 (66.5%) met inclusion criteria. A breakdown of those not meeting inclusion criteria is shown in Table 4.

**Table 4:** Breakdown of inclusion metrics by criterion.

Criterion	Met n (%)	Not met n (%)
**POC HbA**_**1c**_ **64–125 mmol/mol**	464 (69.2)	207 (30.8)
**Age 18+**	671 (100)	0 (0)
**Type 1 or type 2 diabetes**	642 (95.7)	29 (4.3)
** Type 1**	265 (39.5)	
** Type 2**	377 (56.2)	
** Type 3c**		20 (3.0)
** Gestational**		1 (0.1)
** MODY**		1 (0.1)
** Undetermined**		7 (1.0)

**HbA**_**1c**_ **= glycated haemoglobin; MODY = maturity-onset diabetes of the young; POC = point-of-care.**

One-hundred and nine (16.2%) of those screened were eligible for study entry. Reasons for exclusion are shown in Fig 1. Patients at high risk of CV events (Table 5) were excluded due to potentially increased risks associated with adjusting glycated haemoglobin targets in this group.

**Table 5:** Breakdown of patients meeting inclusion criteria screened for risk of cardiovascular events.

Risk factor for CV event	n (%)	Additional risk factors for CV disease	n (%)
Previous MI	24 (4.0)	Dyslipidaemia	64 (9.5)
Previous CVA (stroke or TIA)	20 (3.0)	Two or more elevated urinary ACR	74 (11.0)
Previous coronary artery revascularisation	31 (4.6)	LVH on ECG or echocardiogram	4 (0.6)
Previous or current diagnosis of heart failure	24 (3.6)	Diagnosis of hypertension	215 (32)
50% or more stenosis of coronary, carotid or lower limb extremities on imaging	18 (2.7)	Current smoker	57 (8.5)
Angina with ischaemic changes on ECG at rest or on exertion	5 (0.7)	BMI of 32 kg/m^2^ or more	166 (24.7)
Presence of ASCVD (any one of the above)	81 (12.1)	Two or more additional risk factors for cardiovascular disease	174 (25.9)
At risk of CV events			213 (47.9%)

**ASCVD = atherosclerotic cardiovascular disease; BMI = body mass index; CVA = cerebrovascular event; CV = cardiovascular; ECG = electrocardiogram; LVH = left ventricular hypertrophy.**

**Fig 3:**
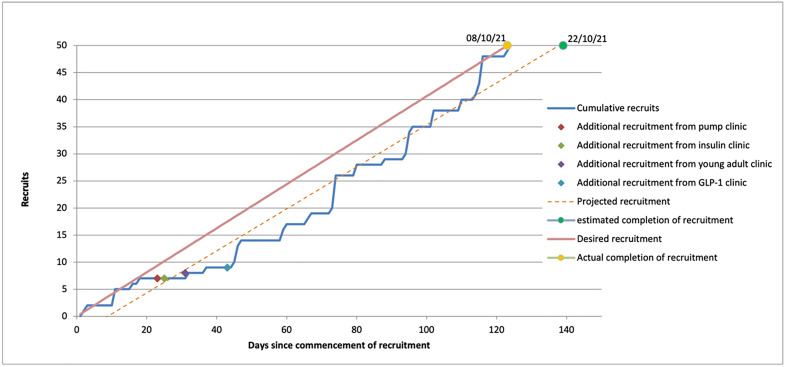
Cumulative recruitment over the 4-month recruitment period.

The required sample was successfully randomised over a 4-month period, with an uptake of 45.9% of those who were eligible to participate. Twenty-seven participants were randomised to Group A, with 23 participants being randomised to Group B. Active monitoring of recruitment rates during the recruitment period noted projected recruitment was below target. Screening of additional participants during the recruitment period allowed for increased recruitment. The graph in Fig 3 demonstrates actual recruitment rates were within the anticipated target. Of those recruited, withdrawal rate was 34% (n = 17), with reasons for withdrawal included in the CONSORT flow chart (Fig 1). Thirty-three adults with diabetes completed the study processes, with 19 participants in Group A, and 14 participants in Group B. Non-completers were noted to be significantly younger (Mdn = 42, IQR = 26) versus completers (Mdn = 51, IQR = 17), U = 176.5, p = .033. No associations were detected between non-completion and gender, diabetes-type, diabetes duration, baseline health status, baseline distress, baseline self-care, baseline wellbeing, baseline self-efficacy, intervention group or index of multiple deprivation (IMD) decile.

Response rate for the PROs was determined on a per-item basis for each questionnaire and each participant. Questionnaire response rate overall was 97.3% in the study completers, or 70.8% if including participants who withdrew. Response rates were similar between groups A (99.7%, or 73.8% when including withdrawers) and B (94.2%, or 67.3% when including withdrawers).

### Glycaemic control

As with all preliminary outcomes reported in this study, caution should be used in interpreting these results. The impact of relaxed (Group A) or intensified (Group B) glycated haemoglobin targets on participants subsequent glycated haemoglobin readings were evaluated.

Where point-of-care glycated haemoglobin was measured in the completers (n = 31, outliers removed, 62%), mean (SD) reduction in glycated haemoglobin was 2.8 (95% CI.7 − 5.0, p = .012) from 69.4 (15.7) at 3 months to 66.6 (16.7) mmol/mol at 6 months. In group A (n = 18, outliers removed), mean glycated haemoglobin improved non-significantly by 2.9 (95% CI −.4 − 6.2, p = .084) from 63.7 (13.6) to 60.8 (13.6) mmol/mol and in group B (n = 13, outliers removed) by 2.8 (95% CI −.2 − 5.7, p = .065) from 77.4 (15.2) to 74.6 (17.7) mmol/mol. Glycated haemoglobin improved to a similar extent in both those with relaxed (group A) or intensified (group B) targets. An independent samples t-test was conducted to compare improvement in glycated haemoglobin in group A and B. There was not a significant difference in improvement in glycated haemoglobin between group B (M = 2.8, SD = 4.9) and group A (M = 2.9, SD = 6.7), t (29)=−.055, p = .957.

### Health-related quality of life

Health status according to the EQ-5D-5L questionnaire outputs two values:

- EQ-5D-5L index, a value between −.594 and 1.000 for the UK with higher values indicating a better health state.
- EQ-VAS, a number derived from a visual analogue scale between 0 (worst health) and 100 (best health).

Minimum clinically important difference (MCID) for EQ-5D-5L index scores is reported in various papers as between 0.030 and 0.074 depending on the condition and country value set used [51–55]. For the UK value set, an MCID of 0.037 is suggested as the smallest detectable change in health-related quality of life according to the EQ-5D-5L that is recognised and valued by the patient [52,53].

At 6 months, 33/50 (66%) of participants completed baseline and endpoint EQ-5D-5L questionnaires. Overall, in the 33 completers, median (IQR) EQ-5D-5L index score reduced from.819 (.285) at 3 months to.795 (.355) at 6 months. In group A completers (n = 19), median EQ-5D-5L index score did not change at 3 months,.837 (.248), and 6 months,.837 (.250). In group B completers (n = 14), median EQ-5D-5L index score reduced from.759 (.508) to.754 (.337) with the Wilcoxon Signed-Ranks Test indicating that there was no significant difference in baseline and endpoint scores, Z = 19, p = .678. Because the data were skewed, a Wilcoxon Signed-Ranks Test was run and the output indicated that overall, endpoint EQ-5D-5L index scores were not significantly lower than baseline, Z = 155.5, p = .875. The Mann-Whitney U test indicated that baseline EQ-5D-5L index scores for group A (Mdn = .837) were not significantly different from those in group B (Mdn = .759); U = 93.0, p = .152 and endpoint EQ-5D-5L index scores for group A (Mdn = .837) were not significantly different from those in group B (Mdn = .754); U = 92.5, p = .142. Because ΔEQ-5D-5L index scores were not skewed, the independent samples t-test was used to compare delta values between groups. The test indicated that delta values for group A (M = −.030, SD = .123) were not significantly different from those in group B (M = .016, SD = .160); t(29)=−.900, p = .375.

At 6 months, overall median EQ-VAS score improved from 75 (20) to 80 (21) with the Wilcoxon Signed-Ranks Test indicating that endpoint scores were not significantly higher than baseline, Z = 184.5, p = .818. In group A completers, median EQ-VAS score did not change at 3 months, 80 (20), and 6 months, 80 (15). In group B completers, median EQ-VAS score improved from 68 (20) at 3 months to 73 (37) at 6 months with the Wilcoxon Signed-Ranks Test indicating that endpoint scores were not significantly higher than baseline, Z = 39, p = .590. The Mann-Whitney U test indicated that baseline EQ-VAS scores for group A (Mdn = 80) were not significantly different from those in group B (Mdn = 68); U = 80.5, p = .055, and endpoint EQ-VAS scores for group A (Mdn = 80) were not significantly different from those in group B (Mdn = 73); U = 80.0, p = .055. Because ΔEQ-VAS scores (n = 31, outliers removed) were not skewed, the independent samples t-test was used to compare delta values between groups. The test indicated that delta values for group A (M = −1.65, SD = 7.8) were not significantly different from those in group B (M = −1.93, SD = 12.9); t(29)=.075, p = .941.

The independent samples t-test was used to compare EQ-5D-5L index delta values between those with type 1 diabetes and those with type 2. The test indicated that delta values for people with type 1 diabetes (M = −.073, SD = .139) were significantly different from those with type 2 (M = .041, SD = .145), mean difference.114 (95% CI.006–.222, p = .038). Further analysis demonstrated no other significant between-group difference when considering gender and whether participants achieved the HbA_1c_ target set.

### Diabetes-related distress

Where diabetes distress was measured in completers (n = 33, 66%), median (IQR) PAID score reduced from 22.5 (26.3) at 3 months to 18.1 (29.4) at 6 months. Because the data were skewed, a Wilcoxon Signed-Ranks Test was run and the output indicated that endpoint PAID scores were significantly lower than baseline, Z = 381.5, p = .009. In group A completers (n = 19), median PAID score reduced from 17.5 (26.3) at 3 months to 10.0 (23.8) at 6 months. The Wilcoxon Signed-Ranks Test indicated that endpoint PAID scores for group A were significantly lower than baseline, Z = 137, p = .025. In group B completers (n = 14), median PAID score reduced from 28.8 (26.3) at 3 months to 24.4 (39.7) at 6 months. The Wilcoxon Signed-Ranks Test indicated that endpoint PAID scores for group B were not significantly lower than baseline, Z = 66, p = .152. The Mann-Whitney U test indicated that baseline PAID scores for group A (Mdn = 17.5) were not significantly different from those in group B (Mdn = 28.8); U = 90.0, p = .123 and endpoint PAID scores for group A (Mdn = 10.0) were not significantly different from those in group B (Mdn = 24.4); U = 102.0, p = .271.

Change in PAID score distributions were not skewed. The independent samples t-test was performed to compare change in PAID score between groups A and B. There was no significant difference in delta values for group A (M = −5.5, SD = 9.6) when compared to group B (M = −4.2, SD = 12.2); t(31)=−.334, p = .741.

### Wellbeing (W-BQ 12)

#### Wellbeing (general).

Overall median (IQR) general wellbeing increased by 3.0 from 20.0 (15.0) at 3 months to 23.0 (11.0) at 6 months with the Wilcoxon Signed-Ranks Test indicating that there was no significant difference in baseline and endpoint scores, Z = −.943, p = .346.

In group A completers (n = 19), median (IQR) general wellbeing increased by 2.0 from 21.0 (14) to 23.0 (10) with the Wilcoxon Signed-Ranks Test indicating that there was no significant difference in baseline and endpoint scores, Z = −.228, p = .820. In group B completers, (n = 14), median (IQR) general wellbeing increased by 5.5 from 16.5 (15) to 22.0 (16) with the Wilcoxon Signed-Ranks Test indicating that there was no significant difference in baseline and endpoint scores, Z = −1.102, p = .270. The Mann-Whitney U test indicated that baseline general wellbeing scores for group A (Mdn = 21.0, IQR = 14) were not significantly different from those in group B (Mdn = 16.5, IQR = 15); U = 90.5, p = .121, and endpoint general wellbeing scores for group A (Mdn = 23.0, IQR = 10) were not significantly different from those in group B (Mdn = 22.0, IQR = 16); U = 122.0, p = .688.

Additionally, after removal of outliers, the Mann-Whitney U test indicated that change in general wellbeing scores for group A (Mdn = 0.0) were not significantly different from those in group B (Mdn = −1.0); U = 97.0, p = .746.

There was no statistically significant change in general wellbeing delta values when analysing by gender.

Multiple linear regression analysis was performed to evaluate for confounders with change in the W-BQ12 general wellbeing score (Δgeneral wellbeing) as the dependent variable and potential confounders as independent variables modelled. It was found that none of the confounding variables significantly predicted change in W-BQ12 general wellbeing score (F[5,27] = 1.322, p = ns).

#### Wellbeing (positive).

Overall mean (SD) PWB score increased by.2 (95% CI −.8–1.1, p = .750) from 6.5 (2.6) at 3 months to 6.7 (2.7) at 6 months. In group A completers (n = 19), mean PWB reduced by.4 (95% CI −.9–1.6, p = .546) from 7.0 (2.5) to 6.6 (2.8). In group B completers (n = 14), mean PWB increased by.8 (95% CI **−**.7 − 2.5, p = .268) from 5.9 (2.6) to 6.7 (2.8).

The independent samples t-test was performed to compare change in PWB score between groups A and B. There was no significant difference in delta values for group A (M = −.4, SD = 2.6) when compared to group B (M = .9, SD = 2.8); t(31)=−1.300, p = .203.

When comparing outcomes by gender, female (n = 15) PWB improved to a significantly greater extent when compared to males (n = 18), with a mean difference of 2.0 (95% CI.2–3.9, p = .028).

Multiple linear regression analysis was performed to evaluate for confounders with change in the W-BQ12 positive wellbeing score (Δpositive wellbeing) as the dependent variable and potential confounders as independent variables modelled. It was found that none of the confounding variables significantly predicted change in W-BQ12 positive wellbeing score (F[5,27] = 2.25, p = ns).

#### Wellbeing (negative).

Where NWB was measured in the completers (n = 33, 66%), overall median (IQR) score reduced by 1.0 from 3.0 (6) at 3 months to 2.0 (4) at 6 months with the Wilcoxon Signed-Ranks Test indicating that there was no significant difference in baseline and endpoint scores, Z = −1.712, p = .087.

In group A completers (n = 19), median (IQR) NWB increased by 1.0 from 1.0 (5) to 2.0 (4) with the Wilcoxon Signed-Ranks Test indicating that there was no significant difference in baseline and endpoint scores, Z = −1.758, p = .079. In group B completers, (n = 14), median (IQR) NWB reduced by 0.5 from 3.5 (6) to 3.0 (5) with the Wilcoxon Signed-Ranks Test indicating that there was no significant difference in baseline and endpoint scores, Z = −.517, p = .607. The Mann-Whitney U test indicated that baseline NWB scores for group A (Mdn = 1.0, IQR = 5) were not significantly different from those in group B (Mdn = 3.5, IQR = 6); U = 101.5, p = .243, and endpoint NWB scores for group A (Mdn = 2.0, IQR = 4) were not significantly different from those in group B (Mdn = 3.0, IQR = 5); U = 91.0, p = .117.

Additionally, the Mann-Whitney U test indicated that change in NWB scores for group A (Mdn = 0.0) were not significantly different from those in group B (Mdn = 0.0); U = 117.5, p = .559.

There was no statistically significant change in NWB delta values when analysing by gender.

Multiple linear regression analysis was performed to evaluate for confounders with change in the W-BQ12 positive wellbeing score (Δpositive wellbeing) as the dependent variable and potential confounders as independent variables modelled. It was found that none of the confounding variables significantly predicted change in W-BQ12 positive wellbeing score (F[5,27] = 2.25, p = ns).

#### Wellbeing (energy).

Overall mean (SD) energy score increased by.6 (95% CI −.3–1.5, p = .207) from 5.1 (3.0) at 3 months to 5.7 (3.0) at 6 months. In group A completers (n = 19), mean energy score reduced by.1 (95% CI −.8–1.0, p = .771) from 5.8 (2.8) to 5.7 (2.5). In group B completers (n = 14), mean energy score increased by 1.5 (95% CI −.3–3.3, p = .092) from 4.1 (3.1) to 5.6 (3.6).

The independent samples t-test was performed to compare change in energy score between groups A and B. With outliers removed, there was no significant difference in delta values for group A (M = −.1, SD = 1.8) when compared to group B (M = .6, SD = 2.2); t(29)=−.974, p = .338.

There was no statistically significant change in energy delta values when analysing by gender.

Multiple linear regression analysis was performed to evaluate for confounders with change in W-BQ12 energy scores (Δenergy) as the dependent variable and potential confounders as independent variables modelled. It was found that none of the confounding variables significantly predicted change in W-BQ12 energy score (F[5,27] =.36, p = ns).

### Diabetes-related psychosocial self-efficacy (managing psychosocial aspects of diabetes)

#### Managing psychosocial aspects of diabetes.

Where diabetes-related psychosocial self-efficacy was measured in completers (n = 33), mean (SD) improvement in managing psychosocial aspects of diabetes was.27 (95% CI.02 − .51, p = .032), from 3.71 (.74) at 3 months to 3.97 (.48) at 6 months. In group A completers (n = 19), mean (SD) improvement was.10 (95% CI −.15 − .35, p = .420), from 3.89 (.57) to 3.99 (.52). In group B completers (n = 14), mean (SD) improvement was.49 (95% CI.02 − .96, p = .041), from 3.45 (.88) to 3.94 (.42).

The independent samples t-test was performed to compare change in managing psychosocial aspects of diabetes score between groups A and B. After removing outliers, there was no significant difference in delta values for group A (M = .10, SD = .52) and group B (M = .26, SD = .61); t(29)=−.778, p = .443. These results suggest that the study intervention had no impact on managing psychosocial aspects of diabetes scores.

Multiple linear regression analysis was performed to evaluate for confounders with change in DES-LF managing psychosocial aspects of diabetes scores (ΔDES-LF managing psychosocial aspects of diabetes) as the dependent variable and potential confounders as independent variables modelled. It was found that none of the confounding variables significantly predicted change in DES-LF managing psychosocial aspects of diabetes score (F[5,27] =.75, p = ns).

### Assessing dissatisfaction and readiness for change

Overall scores for assessing dissatisfaction and readiness to change (n = 33, 66%) did not change, with median (IQR) scores at 3 months, 3.89 (.61), and 6 months, 3.89 (.78). In group A completers (n = 19), median (IQR) scores improved by.11 from 3.78 (.67) to 3.89 (.89) with the Wilcoxon Signed-Ranks Test indicating there was no significant difference in baseline and endpoint scores, Z = −1.647, p = .100. In group B completers (n = 14), median (IQR) scores improved by.11 from 3.89 (.53) to 4.00 (.72) with the Wilcoxon Signed-Ranks Test indicating that there was a significant difference in baseline and endpoint scores, Z = −2.210, p = .027. The Mann-Whitney U test indicated that baseline scores for group A (Mdn = 3.78, IQR = .67) were not significantly different from those in group B (Mdn = 3.89, IQR = .53); U = 132.5, p = .985, and endpoint scores for group A (Mdn = 3.89, IQR = .89) were not significantly different from those in group B (Mdn = 4.00, IQR = .72); U = 114.5, p = .497.

After removal of outliers, change in assessing dissatisfaction and readiness to change score distributions were not skewed. The independent samples t-test was performed to compare delta values between groups A and B. There was no significant difference in scores for group A (M = .25, SD = .39) and group B (M = .26, SD = .40); t(29)=−.127, p = .900. These results suggest that the study intervention had no impact on assessing dissatisfaction and readiness to change scores.

Multiple linear regression analysis was performed to evaluate for confounders with change in DES-LF assessing dissatisfaction and readiness for change scores (ΔDES-LF assessing dissatisfaction and readiness for change) as the dependent variable and potential confounders as independent variables modelled. It was found that none of the confounding variables significantly predicted change in DES-LF assessing dissatisfaction and readiness for change score (F[5,27] =.81, p = ns).

### Setting and achieving diabetes goals

After removal of outliers, where setting and achieving diabetes goals was measured in completers (n = 31), mean (SD) score improved by.16 (95% CI −.09 − .41, p = .214) from 3.90 (.56) to 4.06 (.51). In group A completers (n = 18), mean (SD) improvement was.10 (95% CI-.21 − .41, p = .511) from 4.06 (.56) to 4.16 (.46). In group B completers (n = 13), mean (SD) improvement was.23 (95% CI −.23 − .69, p = .296) from 3.68 (.49) to 3.92 (.55).

The independent samples t-test was performed to compare change in setting and achieving diabetes goals score between groups A and B. There was no significant difference in delta values for group A (M = .10, SD = .63) and group B (M = .23, SD = .76); t(29)=−.526, p = .603. These results suggest that the study intervention had no impact on setting and achieving diabetes goals scores.

Multiple linear regression analysis was performed to evaluate for confounders with change in DES-LF setting and achieving diabetes goals scores (ΔDES-LF setting and achieving diabetes goals) as the dependent variable and potential confounders as independent variables modelled. It was found that none of the confounding variables significantly predicted change in DES-LF setting and achieving diabetes goals score (F[5,27] =.80, p = ns).

### Overall self-efficacy

After removal of outliers, where overall self-efficacy was measured in completers (n = 31), mean (SD) score improved by.25 (95% CI.09 − .41, p = .004) from 3.78 (.46) to 4.03 (.44). In group A completers (n = 18), mean (SD) improvement was.22 (95% CI.04 − .40, p = .018) from 3.84 (.46) to 4.06 (.46). In group B completers (n = 13), mean (SD) improvement was.29 (95% CI −.05 − .63, p = .086) from 3.70 (4.7) to 3.99 (.44).

The independent samples t-test was performed to compare change in overall score between groups A and B. There was no significant difference in delta values for group A (M = .22, SD = .36) and group B (M = .29, SD = .56); t(29)=−.419, p = .678.

Multiple linear regression analysis was performed to evaluate for confounders with change in overall self-efficacy scores (ΔDES-LF overall self-efficacy) as the dependent variable and potential confounders as independent variables modelled. It was found that none of the confounding variables significantly predicted change in overall self-efficacy score (F[5,27] =.77, p = ns).

### Body mass index and blood pressure

Where BMI was measured in completers, (n = 31, 62%), mean (SD) reduction in BMI was.4 (95% CI −.16 − 1.0, p = .151) from 31.0 (6.4) to 30.6 (6.3) kg/m2. In group A (n = 18), mean BMI reduced by.3 (95% CI −.3 − .7, p = .387) from 30.5 (6.0) to 30.2 (5.9). In group B (n = 11), mean BMI reduced by.8 (95% CI −.7 − 2.3, p = .267) from 31.9 (7.1) to 31.1 (7.1).

Where blood pressure was measured in completers, (n = 20), mean (SD) reduction in SBP was 9.2 (95% CI 3.0 − 15.3, p = .006) from 138.4 (19.1) to 129.3 (21.0) and mean reduction in DBP was 1.9 (95% CI −2.0 − 5.7, p = .325) from 79.0 (9.1) to 77.1 (9.0). In group A (n = 12), mean reduction in SBP was 8.8 (95% CI.3 − 17.4, p = .044) from 137.4 (18.1) to 128.6 (23.9) and mean reduction in DBP was 2.5 (95% CI −1.8 − 6.8, p = .228) from 79.3 (9.1) to76.8 (10.4). In group B (n = 8), mean reduction in SBP was 9.6 (95% CI −1.9 − 21.1, p = .090) from 139.9 (21.8) to 130.3 (17.3) and mean reduction in DBP was.9 (95% CI −7.8 − 9.6, p = .818) from 78.4 (9.8) to 77.5 (6.8).

A Summary of all quantitative results is presented in Table 6, with between group differences and p-values in Table 7.

**Table 6:** Results table demonstrating pre- and post-intervention scores for secondary outcome measures in the completers.

	Group A (n = 19)				Group B (n = 14)				Overall (n = 33)			
	Pre	Post	Difference	p-value	Pre	Post	Difference	p-value	Pre	Post	Difference	p-value
Secondary outcomes	Mean (SD), *Median (IQR)*	Mean (SD), *Median (IQR)*	(95% CI)		Mean (SD), *Median (IQR)*	Mean (SD), *Median (IQR)*	(95% CI)		Mean (SD), *Median (IQR)*	Mean (SD), *Median (IQR)*	(95% CI)	
**POC HbA**_**1c**_	63.7 (13.6)	60.8 (13.6)	−2.9 (−6.2‒.4)	.084^	77.4 (15.2)	74.6 (17.7)	−2.8 (−5.7‒.2)	.065^	69.4 (15.7)	66.6 (16.7)	−2.8 (−5.0 to −.7)	**.012^**
**BMI**	30.5 (6.0)	30.2 (5.9)	−.3 (−.7‒.3)	.387^	31.9 (7.1)	31.1 (7.1)	−.8 (−2.3–.7)	.267^	31.0 (6.4)	30.6 (6.3)	−.4 (−1.0–.2)	.151^
**SBP**	137.4 (18.1)	128.6 (23.9)	−8.8 (−17.4 to −.3)	**.044^**	139.9 (21.8)	130.3 (17.3)	−9.6 (−21.1–1.9)	.090^	138.4 (19.1)	129.3 (21.0)	−9.2 (−15.3–3.0)	**.006^**
**DBP**	79.3 (9.1)	76.8 (10.4)	−2.5 (−6.8–1.8)	.228^	78.4 (9.8)	77.5 (6.8)	−.9 (−9.6–7.8)	.818^	79.0 (9.1)	77.1 (9.0)	−1.9 (−5.7–2.0)	.325^
**EQ-5D-5L – ‘health state’**												
**EQ-5D-5L index score**	*.837 (.248)*	*.837 (.250)*	0	n/a	*.759 (.508)*	*.754 (.337)*	−0.005	.678*	*.819 (.285)*	*.795 (.355)*	−0.024	.875*
**EQ-VAS**	*80 (20)*	*80 (15)*	0	n/a	*68 (20)*	*73 (37)*	5	.590*	*75 (20)*	*80 (21)*	5	.818*
**PAID – ‘distress’**												
**PAID**	*17.5 (26.3)*	*10.0 (23.8)*	−7.5	.025*	*28.8 (26.3)*	*24.4 (39.7)*	−4.4	.152*	*22.5 (26.3)*	*18.1 (29.4)*	−4.4	.009*
**SDSCA – ‘self-care’**												
**General Diet**	4.37 (2.15)	4.11 (2.04)	−0.26 (−1.24–.71)	.576^	3.57 (1.38)	4.11 (1.06)	0.54 (−.13–1.20)	.150^	4.03 (1.88)	4.11 (1.67)	.08 (−.54–.69)	.803^
**Specific Diet**	3.22 (1.44)	3.34 (1.69)	0.12 (−.41–.73)	.558^	3.71 (1.27)	3.89 (1.27)	0.18 (−.51–.86)	.583^	3.60 (1.21)	3.77 (1.35)	.17 (−.24–.58)	.410^
**Exercise**	3.11 (2.45)	2.26 (2.11)	−0.85 (−1.6 to −.8)	**.032^**	3.11 (1.75)	3.82 (1.91)	0.71 (−.24–1.67)	.126^	3.11 (2.15)	2.92 (2.14)	.18 (−.44–.81)	.559^
**Blood glucose** **testing**	*5.00 (3.5)*	*4.00 (4.6)*	−1	.301*	*6.00 (5.0)*	*3.25 (3.3)*	−2.75	.858*	*5.00 (4.5)*	*4.50 (4.8)*	−0.5	.744*
**Foot care**	3.50 (7.0)	3.50 (5.0)	0	n/a	*1.25 (4.0)*	*3.00 (2.6)*	1.75	.592*	3.00 (5.0)	3.00 (2.5)	0	n/a
**W-BQ12 – ‘wellbeing’**												
**Negative wellbeing**	*1.0 (5)*	*2.0 (4)*	1.0	.079*	*3.5 (6)*	*3.0 (5)*	−0.5	.607*	*3.0 (6)*	*2.0 (4)*	1.0	.087*
**Positive wellbeing**	7.0 (2.5)	6.6 (2.8)	−0.4 (−1.6 − .9)	.546^	5.9 (2.6)	6.7 (2.8)	0.8 (**−**.7 − 2.5)	.268^	6.5 (2.6)	6.7 (2.7)	.2 (−.8–1.1)	.750^
**Energy**	5.8 (2.8)	5.7 (2.5)	−0.1 (−.8–1.0)	.771^	4.1 (3.1)	5.6 (3.6)	1.5 (−.3–3.3)	.092^	5.1 (3.0)	5.7 (3.0)	.6 (−.3–1.5)	.207^
**General wellbeing**	*21.0 (14)*	*23.0 (10)*	2.0	.820*	*16.5 (15)*	*22.0 (16)*	5.5	.270*	*20.0 (15.0)*	*23.0 (11.0)*	3.0	.346*
**DES-LF – ‘self-efficacy’**												
**Managing** **psychosocial** **aspects of diabetes**	3.89 (.57)	3.99 (.52)	0.1 (−.15 − .35)	.420^	3.45 (.88)	3.94 (.42)	0.49 (.02 − .96)	**.041^**	3.71 (.74)	3.97 (.48)	.27 (02–.51)	**.032^**
**Assessing** **dissatisfaction and** **readiness to change**	*3.78 (.67)*	*3.89 (.89)*	0.11	.100*	*3.89 (.53)*	*4.00 (.72)*	0.11	.027*	3.89 (.61)	3.89 (.78)	0	n/a
**Setting and** **achieving diabetes** **goals**	4.06 (.56)	4.16 (.46)	0.10 (−.21 − .41)	.511^	3.68 (.49)	3.92 (.55)	0.24 (−.23 − .69)	.296^	3. 90 (.56)	4.06 (.51)	.16 (−.09–.41)	.214^
**Overall score**	3.84 (.46)	4.06 (.46)	0.22 (.04 − .40)	**.018^**	3.70 (4.7)	3.99 (.44)	0.29 (−.05 − .63)	.086^	3.78 (.46)	4.03 (.44)	.25 (.09–.41)	**.004^**

* = p value calculated with Wilcoxon signed-ranks test, ^ = p value calculated with paired sample t-test, significant p-values in bold.

**Dark green boxes denote statistically significant improvement; light green, non-significant improvement; dark red, significant worsening; light red, non-significant worsening.**

**BMI = body mass index, CI = confidence interval, DBP = diastolic blood pressure, DES-LF = diabetes empowerment scale-long form questionnaire, EQ-5D-5L = EuroQoL-5D-5L questionnaire, HbA**_**1c**_ **= point-of-care glycated haemoglobin, IQR = interquartile range, SBP = systolic blood pressure, SD = standard deviation, SDSCA = summary of diabetes self-care activities questionnaire, VAS = visual analogue scale, W-BQ12 = wellbeing questionnaire 12.**

**Table 7:** Results table demonstrating delta values for secondary outcome measures by intervention group.

Secondary outcomes	ΔGroup A – relaxed targets (n = 19)	ΔGroup B – intensified targets (n = 14)	p-value
**Mean (SD), *Median (IQR)***	**Mean (SD), *Median (IQR)***	**p-value**
**POC HbA**_**1c**_	−2.9 (6.7)	−2.8 (4.9)	.957^‡^
**EQ-5D-5L index score**	−.030 (.123)	.016 (.160)	.375^‡^
**EQ-VAS**	−1.6 (7.8)	−1.93 (12.9)	.941^‡^
**PAID**	−5.5 (9.6)	−4.2 (12.2)	.741^‡^
**SDSCA**			
**General Diet**	−.26 (2.0)	.54 (1.2)	.194^‡^
**Specific Diet**	.16 (1.1)	.18 (1.2)	.968^‡^
**Exercise**	−.84 (1.6)	.71 (1.6)	**.010** ^‡^
**Blood glucose testing**	.24 (1.35)	−.11 (2.03)	.563^‡^
**Foot care**	*.00 (1.0)*	*.00 (3.4)*	n/a
**W-BQ12**			
**Negative wellbeing**	*0.0 (3)*	*0.0 (8)*	n/a
**Positive wellbeing**	−.4 (2.6)	.9 (2.8)	.203^‡^
**Energy**	−.1 (1.8)	.6 (2.2)	.338^‡^
**General wellbeing**	*0.0 (7)*	*−1.0 (6)*	.746^†^
**DES-LF**			
**Managing psychosocial aspects of diabetes**	.10 (.52)	.26 (.61)	.443^‡^
**Assessing dissatisfaction and readiness to change**	.25 (.39)	.26 (.40)	.900^‡^
**Setting and achieving diabetes goals**	.10 (.63)	.23 (.76)	.603^‡^
**Overall score**	.22 (.36)	.29 (.56)	.678^‡^

† ** = p value calculated with Mann-Whitney U,** ‡ ** = p value calculated with independent sample t-test**

**DES-LF = diabetes empowerment scale-long form questionnaire, EQ-5D-5L = EuroQoL-5D-5L questionnaire, HbA**_**1c**_ **= point-of-care glycated haemoglobin, IQR = interquartile range, SD = standard deviation, SDSCA = summary of diabetes self-care activities questionnaire, VAS = visual analogue scale, W-BQ12 = wellbeing**

### Confounders

Evaluation for confounding variables (gender, age, diabetes duration, IMD decile, BMI) with multiple linear regression stepwise modelling revealed only one out of twenty-five secondary outcomes listed in Table 7 were potentially affected by these confounders. For the change in SDSCA foot care score, multiple linear regression analysis resulted in a model suggesting BMI was a significant predictor of SDSCA foot care scores with an inverse relationship – as BMI increased, foot care activity decreased (β = −.60, t[32] = −3.82, p < .01).

### Semi-structured interviews: participant experiences

Semi-structured interviews were used to extract detailed narratives from participants on pre-determined topics of interest; perceptions of the use of individualised glycated haemoglobin targets, diabetes and wellbeing, and acceptability of study processes. All 50 participants in the feasibility study were eligible. Fourteen participants were selected for interview.

Interview timing was scheduled in advance to ensure participants and the researcher were able to coordinate a distraction-free environment. All interviews took place via telephone and were recorded using a password-protected digital Dictaphone. Written, informed consent was obtained prior to interview commencement, including consent for use of anonymised quotes on result publication. Following introductions and confirming participant identity, an interview topic guide (Supporting information S3 File) was used to explore themes of interest and to standardise topics covered. As described in greater depth in the protocol, a semi-structured interview technique was used to provide deeper understanding of topics covered using clarification and follow-up questioning. Interviews were between 18 and 58 minutes in duration (Table 8).

**Table 8:** Characteristics of participants completing interview and free-text responses to an end-of-study acceptability survey, including baseline values from psychometric questionnaires.

Participant number	Randomisation group (feasibility study)	Age (years)	Gender	Diabetes type	Diabetes duration (years)	IMD decile	Interview duration (minutes)	HbA_1c_ (mmol/mol)	EQ-5D-5L INDEX	EQ-VAS	SDSCA general diet	SDSCA exercise	SDSCA blood glucose testing	PAID	W-BQ12 NWB	W-BQ12 PWB	W-BQ12 energy	W-BQ12 overall wellbeing	DES-LF psychosocial*	DES-LF dissatisfaction^†^	DES-LF goals^‡^	DES-LF overall self-efficacy
P1	A	70	Male	Type 2	18	1	29	52	1.000	75	5.5	7	5.5	18.75	0.0	9.0	9.3	30.3	3.78	3.56	4.00	3.79
P2	A	55	Male	Type 2	14	1	24	62	0.750	70	3	3.5	7	60	5.0	3.0	4.0	14.0	3.44	3.44	3.50	3.46
P3	A	47	Male	Type 1	16	8	52	76	1.000	90	7	7	7	38.75	0.0	9.0	8.0	29.0	4.00	4.22	4.00	4.07
P4	A	41	Male	Type 1	0.12	3	44	43	1.000	85	4.5	0.5	3.5	15	0.0	5.0	7.0	24.0	4.00	3.33	3.60	3.64
P5	B	64	Female	Type 2	24	1	31	73	0.710	70	6	3	7	22.5	0.0	10.0	8.0	30.0	4.11	4.11	4.40	4.21
P6	A	41	Male	Type 1	14	8	24	65	0.795	70	0	4.5	7	26.25	0.0	11.0	7.0	30.0	4.44	4.11	3.70	4.07
P7	B	46	Female	Type 2	4	6	30	87	0.249	25	3.5	0	7	48.75	6.0	2.0	2.0	10.0	3.00	4.44	2.50	3.29
P8	B	64	Male	Type 2	10	1	19	–	–	–	–	–	–	–	–	–	–	–	–	–	–	–
P9	B	22	Male	Type 1	3.5	6	42	110	0.750	80	2	5	5	41.25	4.0	6.0	5.0	19.0	3.78	3.78	4.00	3.86
P10	A	46	Male	Type 2	15	1	34	58	0.768	75	6	3.5	4	31.25	4.0	5.0	3.0	16.0	3.56	4.11	3.70	3.79
P11	A	22	Male	Type 1	10	1	49	66	1.000	80	6.5	1.5	7	5	1.0	9.0	6.0	26.0	4.56	4.67	4.70	4.64
P12	A	27	Male	Type 1	13	3	58	76	0.721	70	0	0.5	5	28.75	6.0	5.0	4.0	15.0	3.89	4.11	3.50	3.82
P13	A	20	Male	Type 1	9	3	18	54	0.879	90	6	5.5	6.5	8.75	0.0	9.0	8.0	29.0	4.78	4.22	4.30	4.43
P14	B	49	Female	Type 2	6	1	42	53	0.768	65	2.5	0	2.5	22.5	1.0	4.0	2.0	17.0	3.11	3.89	3.60	3.54

**DES-LF = diabetes empowerment scale – long form; EQ-5D-5L = EuroQoL-5D health related quality of life questionnaire; EQ-VAS = EuroQoL Visual Analogue Scale (overall health state); HbA**_**1c**_ **= glycated haemoglobin; IMD = index of multiple deprivation; mmol/mol = millimoles per mole; NWB = negative wellbeing; PAID = problem areas in diabetes; PWB = positive wellbeing; SDSCA = summary of diabetes self-care activities; W-BQ12 = wellbeing questionnaire (12-item).**

* managing psychosocial aspects of diabetes

**† assessing dissatisfaction and readiness for change**

**‡ setting and achieving diabetes goals**

**Cell results for PROs in each column are colour coded with conditional formatting rules, with better values being green, intermediate values yellow to orange, and worse values being red.**

Interviews with participants explored study feasibility alongside acceptability exploring psychological and physical aspects of their diabetes self-management. Analysed transcript data was used to gain a greater understanding of quantitative data outcomes.

Transcript analysis demonstrated study processes, study documentation, time commitments and use of questionnaires in the study were acceptable. When describing specific factors motivating them towards achieving their HbA_1c_ target, participants commented on the beneficial impact of having a goal or target HbA_1c_ to strive for, potentially reflecting the improvements seen in PROs (albeit non-significant).

“I think it’s really a good idea to have a target because I feel that if you went to your follow-up so many months later and they said, “well, you’re near that target now” I think it would give you more incentive to keep going.”—Type 2, female, 64, group B

Interviews also explored psychological and physical health aspects of diabetes, with many participants commenting on the burden of diabetes self-management and coping strategies they have developed over time. These experiences echo the high degree of diabetes-related distress seen at baseline in our study.

“My diabetes is like a backpack—it just sits on my back.”—Type 1, male, 27, group A

Findings from interviews with study participants demonstrate considerable crossover between quantitative outcomes and participants’ experiences in the study, both in terms of study feasibility and PROs.

Three main themes emerged from these data: study feasibility, glycated haemoglobin targets, and diabetes and wellbeing. Participants discussed factors which aided their decision to consent to participate in the study including face-to-face interaction with the researcher with the opportunity to ask questions prior to consent, and whether the research aims aligned with their ideology. Participants also discussed study retention, with reasons for continued engagement with the research being perceived health benefits, increased mood, additional healthcare interaction, and personal interest in the research topic. Participants discussed the use of glycated haemoglobin targets as part of their diabetes care, with many acknowledging limited understanding of its utility prior to study entry. With additional understanding brought motivation to achieve their target. Motivators were either circumstantial (e.g., the need to reduce HbA_1c_ before surgery) or more general (e.g., to reduce future risk of diabetes complications). Having targets that were achievable in order to maintain motivation for improved self-management emerged as a recurrent theme in interviews. Specific demotivators were numerous, highlighting the persistent effort often required to maintain good levels of self-management in diabetes. Experiences were widely varied, with some participants noting minimal impacts of diabetes on their wellbeing and others noting life-changing psychological, physical or social effects.

Barriers to participation were evaluated in participants declining entry into the feasibility study, highlighting important considerations for adjustment of future protocols in enhancing recruitment and retention of participants. Four main themes emerged during data analysis: barriers to participation, facilitators to participation, treatment targets, and diabetes and wellbeing. Person-specific barriers, such as the presence of disability, were identified by participants alongside study-specific barriers, such as the requirement to attend multiple study visits over a period. Potential facilitators were also discussed, with participants highlighting the importance of research visit flexibility alongside acknowledging the type of research impacted their decision to participate. Views on the themes of ‘treatment targets in diabetes’ and ‘diabetes and wellbeing’ corroborated with the findings of the interviews presented those consenting to take part in the quantitative study components, enhancing data credibility.

### Semi-structured interviews: healthcare professional experiences

In total, eight healthcare professionals were recruited for interview, following the interview topic guide (Supporting information S4 File) to explore themes of interest and to standardise topics covered. Baseline characteristics are noted in Table 9. Participants were recruited from a range of professional backgrounds directly involved in the care of people with diabetes. To maintain participant confidentiality, participants were assigned a unique identifying code (Table 9).

**Table 9:** Participant characteristics.

Participant number	Role	Interview duration
H1	Dietitian	35
H2	Nurse	30
H3	Nurse	52
H4	Nurse	27
H5	Consultant Physician	24
H6	Dietitian	45
H7	Nurse	17
H8	Specialist Trainee (doctor)	36

Interviews explored themes of diabetes management, and diabetes and wellbeing. Findings from analysis of interview transcripts a high degree of crossover with patient experiences, indicating a great degree of empathy and understanding for their patients. Interviews noted the importance of a positive and encouraging narrative in patient consultations, setting achievable goals to motivate improved self-management.

Interviews noted the importance of the doctor-patient relationship in achieving trust and motivating patients.

H5: “The relationship between the DSN, the doctor and the patient, it just makes a huge difference. They have to trust you. And they have to feel confident ask any question that they have.”

Diabetes healthcare professionals demonstrated significant understanding of the impact diabetes and glycated haemoglobin targets have on the wellbeing of their patients, with findings echoing interviews with patients. Two main themes emerged: managing diabetes, and diabetes and wellbeing. Healthcare professionals expressed the importance of discussing HbA_1c_ targets integrated as part of a positive dialogue with patients in order to maintain engagement and motivation. Interviewees noted the importance of targeting HbA_1c_ levels to improve biomedical outcomes for their patients but acknowledged the importance of consideration of the patient perspective where functional or social goals may take precedence. The importance of building rapport with patients was highlighted, especially where psychological issues associated with diabetes were anticipated. Many noted limited training or experience in managing psychological aspects of diabetes care and the limited availability of clinical psychologists with experience in diabetes-specific psychological issues.

## Discussion

This study explored the feasibility of conducting a larger trial to evaluate the impact of setting explicit glycated haemoglobin (HbA_1c_) targets—either intensified or relaxed—on both psychometric and glycaemic outcomes in people with diabetes. In addition to reporting study feasibility, the study captured preliminary effects on PROs and HbA_1c_, and the experiences and perspectives of patients and healthcare professionals regarding study acceptability, wellbeing, and target-setting.

Feasibility data indicated a sufficient population for screening (n = 671), with 109 individuals meeting eligibility criteria (16.2%) and 50 recruited (recruitment rate 45.9%) within the predefined window. Although the dropout rate of 34% was higher than anticipated, it remained within the range reported in a meta-analysis of chronic disease trials, which found an average attrition rate of 43% (95% CI 29–57) [56]. Questionnaire response rate was 70.8% when including withdrawers, aligning with the mean response rate of 70.0% (±18.4) reported in a systematic review of 811 patient surveys [57]. While lower response rates may introduce non-response bias, the literature presents conflicting evidence on this issue [58], and our response rate supports the validity of the findings.

To improve retention in a future definitive trial, protocol amendments should consider strategies identified in the Cochrane review by Gilles *et al.* [59], including telephone reminders, monetary incentives, and open trial designs. Although this study was open by necessity, further enhancements could be made by addressing qualitative feedback on questionnaire length and clarity, and by implementing methods such as telephone follow-up, recorded delivery with return envelopes, and incentives for non-responders.

Acceptability of the intervention was high, with participants motivated to achieve glycaemic goals. Improvements were observed in diabetes-related distress, self-efficacy, and HbA1c, regardless of whether participants received relaxed or intensified targets. Sample size estimates for a future trial, based on mean delta values from key outcomes (Table 7), ranged from 415 to 1,212 per group, assuming α = 0.05 and power = 80%.

Evidence on the psychometric impact of HbA_1c_ targets remains limited, with prior studies relying on surrogate markers rather than direct PRO measurement [60]. Our preliminary findings suggest the need to explore the improvements in HbA1c, systolic blood pressure, diabetes distress, psychosocial management, and self-efficacy at 3 months post-intervention (Table 6) with further research.

Whilst looking at the PRO outcome data with caution, accepting the study was underpowered to demonstrate any statistical significance, health-related quality of life changes were equivocal but warrant further investigation. Unexpectedly, both self-efficacy and distress improved, despite prior literature suggesting no direct relationship between self-efficacy and HbA_1c_ [61]. This raises the possibility that the intervention itself contributed to these improvements. Enhanced understanding of HbA_1c_ targets may have driven self-efficacy gains, consistent with studies showing that increased disease understanding supports better self-management [62–64]. A significant deterioration in EQ-5D-5L index scores was observed among participants with type 1 diabetes compared to those with type 2 (mean difference.114 [95% CI.006–.222, p = .038]), suggesting a potentially different psychological response to target-setting by diabetes type.

Biomedical outcomes (HbA1c, blood pressure, BMI) improved across both groups, with minimal between-group differences. These improvements may reflect increased motivation from goal-setting, enhanced understanding of HbA_1c_ utility, medication adherence, the Hawthorne effect [65], delayed effects of prior medication changes, or chance. Regardless of mechanism, the findings suggest that setting specific glycaemic goals may support improved biomedical outcomes and warrant further investigation.

Qualitative interviews with participants and healthcare professionals were triangulated with quantitative data to enrich interpretation. Thematic analysis revealed strong alignment between quantitative outcomes and participant experiences, particularly regarding acceptability and PROs.

Integration of qualitative and quantitative results presented demonstrate considerable cross-over between PROs and participant experiences of HbA_1c_ target-setting. Many, but not all, PROs aligned with participants’ experiences and views expressed in interviews and free-text survey responses. Preliminary quantitative findings demonstrated benefits to diabetes-related distress, wellbeing and self-management in agreement with qualitative findings of positive experiences described by participants.

Credibility of the findings is enhanced using method triangulation (a combination of both qualitative and quantitative methodologies to check consistency of findings), source triangulation (studying both people with diabetes and healthcare professionals), analytical triangulation (utilising experience from the supervisory team to confirm coding and analytical strategy), and member-checking (testing the validity of participant accounts using probing, clarifying or summarising lines of inquiry).

Participants cited face-to-face interaction, alignment with personal values, perceived health benefits, improved mood, and increased healthcare engagement as motivators for participation and retention. Many reported limited prior understanding of HbA_1c_ targets, with increased knowledge fostering motivation to achieve their goals. Motivators were either circumstantial (e.g., the need to reduce HbA_1c_ before surgery) or more general (e.g., to reduce future risk of diabetes complications). Having targets that were achievable in order to maintain motivation for improved self-management emerged as a recurrent theme in interviews. Specific demotivators were numerous, highlighting the persistent effort often required to maintain good levels of self-management in diabetes. Responses coded to the final theme of ‘diabetes and wellbeing’ were further divided into psychological, physical and social (‘living with diabetes’) sub-themes. Experiences were widely varied, with some participants noting minimal impacts of diabetes on their wellbeing and others noting life-changing psychological, physical or social effects.

Interviews with diabetes healthcare professionals demonstrated significant understanding of the impact diabetes and glycated haemoglobin targets have on the wellbeing of their patients, echoing findings from patient interviews. Two main themes emerged: managing diabetes, and diabetes and wellbeing. Healthcare professionals expressed the importance of discussing HbA_1c_ targets integrated as part of a positive dialogue with patients in order to maintain engagement and motivation. Interviewees noted the importance of targeting HbA_1c_ levels to improve biomedical outcomes for their patients but acknowledged the importance of consideration of the patient perspective where functional or social goals may take precedence. The importance of building rapport with patients was highlighted, especially where psychological issues associated with diabetes were anticipated. Many noted limited training or experience in managing psychological aspects of diabetes care and the limited availability of clinical psychologists with experience in diabetes-specific psychological issues.

## Strengths and limitations

The confidence intervals for these data are wide, reflecting that the study was underpowered to demonstrate statistical significance. By nature of design, the study presented aimed to establish feasibility, and as such was not powered to output statistically significant PRO and biomedical data. Sufficient demographic information has been reported to determine the generalisability of findings, though recruiting from a single centre reduces the overall generalisability. A wide range of participants were recruited in terms of age, diabetes duration, and gender. The population from which study participants were recruited has a higher diabetes prevalence, higher deprivation score, lower life expectancy and higher rates of obesity [66] than the national average in the UK. Additionally, the local population is predominantly white (98.4%) compared with 85.4% nationally [67] resulting in difficulties in recruiting people from ethnic minority groups. On this basis, the findings presented are generalisable to adults with type 1 or type 2 diabetes under secondary care diabetes services in other regions in the UK with comparable demographics. This limitation could be addressed by recruiting from additional centres in England to achieve a broadly representative sample in a future study.

Baseline characteristics between groups A and B demonstrated no significant between-group differences. In reviewing the characteristics of non-completers versus completers, non-completers had a significantly lower age. In many cases for those who dropped out, the reason for drop-out was not obtained. Where captured, reasons for drop-out were work commitments, other health conditions and pandemic-related avoidance of hospital environments. Non-significant higher rates of drop-out were seen in those with stretch targets. This may have introduced attrition bias in that the non-completers with a lower median age may have responded differently to the intervention. As such, between-group differences must be interpreted with caution. Future study protocols should consider strategies to enhance retention of younger participants.

Multiple linear regression was conducted to identify any potential confounding factors (age, gender, diabetes duration, IMD decile, BMI) affecting outcomes. The majority of outcomes were not influenced by potential confounders due to robust randomisation processes. BMI was noted to be a predictor of foot care processes according to the SDSCA. Reduction in foot care in those with increasing BMI may be due to functional limitations (e.g., reduced spine flexibility, limited range of movement of major joints, reduced capacity to hold prolonged fixed postures) seen in adults with obesity [68] limiting foot care processes.

Using a mixed-methods approach, the findings from the quantitative and qualitative study aspects have been considered together as a whole to increase confidence in data trustworthiness. Trustworthiness of the results was determined using Lincoln and Guba’s Evaluative criteria [49], with credibility of the findings being enhanced using method triangulation, source, analytical triangulation, and member-checking.

## Conclusion

This study contributes to the limited evidence base on psychometric outcomes in diabetes and the role of treatment targets, demonstrating the feasibility of conducting a larger, definitive trial with protocol refinements. While preliminary findings suggest that setting explicit glycated haemoglobin (HbA_1c_) targets may be associated with improvements in patient-reported outcomes, biomedical measures, and patient experience, these results must be interpreted cautiously given the small sample size, limited statistical power, and potential biases inherent in a feasibility design.

The study adds novel insights by directly examining PROs such as health-related quality of life, self-care, distress, wellbeing, and self-efficacy in relation to HbA_1c_ target-setting—areas that have previously been underexplored. Importantly, qualitative data highlighted how patient understanding and motivation interacted with quantitative improvements, underscoring the value of integrating patient perspectives into trial design and clinical practice.

Implementation of individualised HbA_1c_ targets should be grounded in shared decision-making between patients and clinicians, balancing risks of future complications with patient preferences utilised [69]. The complex relationship between psychological and physical health observed here—particularly the interplay between self-efficacy, distress, and glycaemic control—suggests that target-setting may influence self-management behaviours, though further research is required to establish causality and long-term impact.

Overall, this feasibility study provides methodological and experiential insights to inform a larger trial, while reinforcing the importance of patient-centred approaches in diabetes care.

## Supporting information

S1 File CONSORT 2010 Checklist. (DOC)

S2 File Approved protocol. (PDF)

S3 File Patient interview topic guide. (PDF)

S4 File Healthcare professional interview topic guide. (PDF)

## References

[1] ORIGIN Trial Investigators, Mellbin LG, Rydén L, Riddle MC, Probstfield J, Rosenstock J, et al. Does hypoglycaemia increase the risk of cardiovascular events? A report from the ORIGIN trial. Eur Heart J. 2013;34(40):3137–44. doi: 10.1093/eurheartj/eht332 23999452

[2] Mukamal KJ, Nesto RW, Cohen MC, Muller JE, Maclure M, Sherwood JB, et al. Impact of diabetes on long-term survival after acute myocardial infarction: comparability of risk with prior myocardial infarction. Diabetes Care. 2001;24(8):1422–7. doi: 10.2337/diacare.24.8.1422 11473080

[3] Brown FW. Depression and Diabetes. J Clin Psychiatry. 2011;72(08):1159–60. doi: 10.4088/jcp.11bk06921

[4] Egede LE, Nietert PJ, Zheng D. Depression and all-cause and coronary heart disease mortality among adults with and without diabetes. Diabetes Care. 2005;28(6):1339–45. doi: 10.2337/diacare.28.6.1339 15920049

[5] Action to Control Cardiovascular Risk in Diabetes Study Group, Gerstein HC, Miller ME, Byington RP, Goff DC Jr, Bigger JT, et al. Effects of intensive glucose lowering in type 2 diabetes. N Engl J Med. 2008;358(24):2545–59. doi: 10.1056/NEJMoa0802743 18539917 PMC4551392

[6] Lipska KJ, Ross JS, Miao Y, Shah ND, Lee SJ, Steinman MA. Potential overtreatment of diabetes mellitus in older adults with tight glycemic control. JAMA Intern Med. 2015;175(3):356–62. doi: 10.1001/jamainternmed.2014.7345 25581565 PMC4426991

[7] Shorr RI. Incidence and Risk Factors for Serious Hypoglycemia in Older Persons Using Insulin or Sulfonylureas. Arch Intern Med. 1997;157(15):1681. doi: 10.1001/archinte.1997.00440360095010 9250229

[8] Monami M, Vitale V, Lamanna C, Bartoli N, Martelli D, Zannoni S, et al. HbA1c levels and all-cause mortality in type 2 diabetic patients: epidemiological evidence of the need for personalised therapeutic targets. Nutr Metab Cardiovasc Dis. 2013;23(4):300–6. doi: 10.1016/j.numecd.2012.01.003 22633797

[9] Liccini A, Malmstrom TK. Frailty and Sarcopenia as Predictors of Adverse Health Outcomes in Persons With Diabetes Mellitus. J Am Med Dir Assoc. 2016;17(9):846–51. doi: 10.1016/j.jamda.2016.07.007 27569712

[10] Yanagita I, Fujihara Y, Eda T, Tajima M, Yonemura K, Kawajiri T, et al. Low glycated hemoglobin level is associated with severity of frailty in Japanese elderly diabetes patients. J Diabetes Investig. 2018;9(2):419–25. doi: 10.1111/jdi.12698 28556518 PMC5835456

[11] Kalyani RR, Saudek CD, Brancati FL, Selvin E. Association of diabetes, comorbidities, and A1C with functional disability in older adults: results from the National Health and Nutrition Examination Survey (NHANES), 1999-2006. Diabetes Care. 2010;33(5):1055–60. doi: 10.2337/dc09-1597 20185736 PMC2858174

[12] Zoungas S, Patel A, Chalmers J, de Galan BE, Li Q, Billot L, et al. Severe hypoglycemia and risks of vascular events and death. N Engl J Med. 2010;363(15):1410–8. doi: 10.1056/NEJMoa1003795 20925543

[13] Davies MJ, D’Alessio DA, Fradkin J, Kernan WN, Mathieu C, Mingrone G, et al. Management of hyperglycaemia in type 2 diabetes, 2018. A consensus report by the American Diabetes Association (ADA) and the European Association for the Study of Diabetes (EASD). Diabetologia. 2018;61(12):2461–98. doi: 10.1007/s00125-018-4729-5 30288571

[14] Davies MJ, D’Alessio DA, Fradkin J, Kernan WN, Mathieu C, Mingrone G, et al. Correction to: Management of hyperglycaemia in type 2 diabetes, 2018. A consensus report by the American Diabetes Association (ADA) and the European Association for the Study of Diabetes (EASD). Diabetologia. 2019;62(5):873. doi: 10.1007/s00125-019-4845-x 30899969

[15] World Health Organization. Definition, Diagnosis and Classification of Diabetes Mellitus and its Complications. 1999. https://apps.who.int/iris/bitstream/handle/10665/66040/WHO_NCD_NCS_99.2.pdf?sequence=1&isAllowed=y

[16] Liew G, Michaelides M, Bunce C. A comparison of the causes of blindness certifications in England and Wales in working age adults (16–64 years), 1999–2000 with 2009–2010. BMJ Open. 2014;4:4015. doi: 10.1136/bmjopen-2013 PMC3927710 24525390

[17] Noto H, Tsujimoto T, Sasazuki T, Noda M. Significantly Increased Risk of Cancer in Patients with Diabetes Mellitus: A Systematic Review and Meta-Analysis. Endocrine Practice. 2011;17(4):616–28. doi: 10.4158/ep10357.ra 21454235

[18] Ohkuma T, Peters SAE, Woodward M. Sex differences in the association between diabetes and cancer: a systematic review and meta-analysis of 121 cohorts including 20 million individuals and one million events. Diabetologia. 2018;61(10):2140–54. doi: 10.1007/s00125-018-4664-5 30027404 PMC6133170

[19] Cheng G, Huang C, Deng H, Wang H. Diabetes as a risk factor for dementia and mild cognitive impairment: a meta-analysis of longitudinal studies. Intern Med J. 2012;42(5):484–91. doi: 10.1111/j.1445-5994.2012.02758.x 22372522

[20] Chatterjee S, Peters SAE, Woodward M, Mejia Arango S, Batty GD, Beckett N, et al. Type 2 Diabetes as a Risk Factor for Dementia in Women Compared With Men: A Pooled Analysis of 2.3 Million People Comprising More Than 100,000 Cases of Dementia. Diabetes Care. 2016;39(2):300–7. doi: 10.2337/dc15-1588 26681727 PMC4722942

[21] NHS Digital. National Diabetes Audit, 2015-16 Report 2a: Complications and Mortality (Complications of Diabetes). 2016.

[22] NHS Digital. National Diabetes Audit, 2017-18 Care Processes and Treatment Targets short report. 2018. https://files.digital.nhs.uk/E6/369B83/NationalDiabetesAudit2017–18ShortReport%2CCareProcessesandTreatmentTargets.pdf

[23] Mahieu R. ‘We’re not coming from Mars; we know how things work in Morocco!’ How diasporic Moroccan youth resists political socialisation in state-led homeland tours. Journal of Ethnic and Migration Studies. 2017;45(4):674–91. doi: 10.1080/1369183x.2017.1409177

[24] González-Castro TB, Escobar-Chan YM, Fresan A, López-Narváez ML, Tovilla-Zárate CA, Juárez-Rojop IE, et al. Higher risk of depression in individuals with type 2 diabetes and obesity: Results of a meta-analysis. J Health Psychol. 2021;26(9):1404–19. doi: 10.1177/1359105319876326 31532262

[25] Anderson RJ, Freedland KE, Clouse RE, Lustman PJ. The prevalence of comorbid depression in adults with diabetes: a meta-analysis. Diabetes Care. 2001;24(6):1069–78. doi: 10.2337/diacare.24.6.1069 11375373

[26] Egbuonu I, Trief PM, Roe C, Weinstock RS. Glycemic outcomes related to depression in adults with type 1 diabetes. J Health Psychol. 2021;26(6):786–94. doi: 10.1177/1359105319845134 33904320

[27] Ali MK, Bullard KM, Saaddine JB, Cowie CC, Imperatore G, Gregg EW. Achievement of goals in U.S. diabetes care, 1999-2010. N Engl J Med. 2013;368(17):1613–24. doi: 10.1056/NEJMsa1213829 23614587

[28] Berikai P, Meyer PM, Kazlauskaite R, Savoy B, Kozik K, Fogelfeld L. Gain in patients’ knowledge of diabetes management targets is associated with better glycemic control. Diabetes Care. 2007;30(6):1587–9. doi: 10.2337/dc06-2026 17372160

[29] Grant RW, Pabon-Nau L, Ross KM, Youatt EJ, Pandiscio JC, Park ER. Diabetes oral medication initiation and intensification: patient views compared with current treatment guidelines. Diabetes Educ. 2011;37(1):78–84. doi: 10.1177/0145721710388427 21115980 PMC3033981

[30] O’Connor PJ, Crabtree BF, Yanoshik MK. Differences between diabetic patients who do and do not respond to a diabetes care intervention: a qualitative analysis. Fam Med. 1997;29(6):424–8. 9193915

[31] Diabetes Control and Complications Trial Research Group, Nathan DM, Genuth S, Lachin J, Cleary P, Crofford O, et al. The effect of intensive treatment of diabetes on the development and progression of long-term complications in insulin-dependent diabetes mellitus. N Engl J Med. 1993;329(14):977–86. doi: 10.1056/NEJM199309303291401 8366922

[32] UK Prospective Diabetes Study (UKPDS) Group. Intensive blood-glucose control with sulphonylureas or insulin compared with conventional treatment and risk of complications in patients with type 2 diabetes (UKPDS 33). The Lancet. 1998;352(9131):837–53. doi: 10.1016/s0140-6736(98)07019-6 9742976

[33] Ismail-Beigi F, Moghissi E, Tiktin M, Hirsch IB, Inzucchi SE, Genuth S. Individualizing glycemic targets in type 2 diabetes mellitus: implications of recent clinical trials. Ann Intern Med. 2011;154(8):554–9. doi: 10.7326/0003-4819-154-8-201104190-00007 21502652

[34] UKPDS Group. Effect of intensive blood-glucose control with metformin on complications in overweight patients with type 2 diabetes (UKPDS 34). The Lancet. 1998;352(9131):854–65. doi: 10.1016/s0140-6736(98)07037-8 9742977

[35] Westall SJ, Watmough S, Narayanan RP, Irving G, Hardy K. Psychometric and biomedical outcomes of glycated haemoglobin target-setting in adults with type 1 and type 2 diabetes: Protocol for a mixed-methods parallel-group randomised feasibility study. PLoS One. 2022;17(10):e0275980. doi: 10.1371/journal.pone.0275980 36302049 PMC9612465

[36] Westall SJ. Psychometric and biomedical outcomes of setting explicit glycated haemoglobin targets in adults with diabetes: A mixed-methods parallel-group randomised feasibility study. Edge Hill University. 2024. https://research.edgehill.ac.uk/en/studentTheses/psychometric-and-biomedical-outcomes-of-setting-explicit-glycated 10.1371/journal.pone.0275980 PMC9612465 36302049

[37] Hoffmann TC, Glasziou PP, Boutron I, Milne R, Perera R, Moher D, et al. Better reporting of interventions: template for intervention description and replication (TIDieR) checklist and guide. BMJ. 2014;348:g1687. doi: 10.1136/bmj.g1687 24609605

[38] Chan A-W, Tetzlaff JM, Altman DG, Laupacis A, Gøtzsche PC, Krleža-Jerić K, et al. SPIRIT 2013 statement: defining standard protocol items for clinical trials. Ann Intern Med. 2013;158(3):200–7. doi: 10.7326/0003-4819-158-3-201302050-00583 23295957 PMC5114123

[39] Herdman M, Gudex C, Lloyd A, Janssen M, Kind P, Parkin D, et al. Development and preliminary testing of the new five-level version of EQ-5D (EQ-5D-5L). Qual Life Res. 2011;20(10):1727–36. doi: 10.1007/s11136-011-9903-x 21479777 PMC3220807

[40] Polonsky WH, Anderson BJ, Lohrer PA, Welch G, Jacobson AM, Aponte JE, et al. Assessment of diabetes-related distress. Diabetes Care. 1995;18(6):754–60. doi: 10.2337/diacare.18.6.754 7555499

[41] Toobert DJ, Hampson SE, Glasgow RE. The summary of diabetes self-care activities measure: results from 7 studies and a revised scale. Diabetes Care. 2000;23(7):943–50. doi: 10.2337/diacare.23.7.943 10895844

[42] Plowright R, Witthaus E, Bradley C. Evaluating the 12-item Well-being Questionnaire for use in multinational trials. Qual Life Res. 1999;8:650.

[43] Anderson RM, Funnell MM, Fitzgerald JT, Marrero DG. The Diabetes Empowerment Scale: a measure of psychosocial self-efficacy. Diabetes Care. 2000;23(6):739–43. doi: 10.2337/diacare.23.6.739 10840988

[44] IBM Corp. IBM SPSS Statistics for Windows. Armonk, N.Y., USA: IBM Corp. 2017.

[45] NIHR. Feasibility and pilot studies: a guide for NIHR Research Design Service advisors. 2016.

[46] Bell ML, Whitehead AL, Julious SA. Guidance for using pilot studies to inform the design of intervention trials with continuous outcomes. Clin Epidemiol. 2018;10:153–7. doi: 10.2147/CLEP.S146397 29403314 PMC5779280

[47] Gale NK, Heath G, Cameron E, Rashid S, Redwood S. Using the framework method for the analysis of qualitative data in multi-disciplinary health research. BMC Med Res Methodol. 2013;13:117. doi: 10.1186/1471-2288-13-117 24047204 PMC3848812

[48] QSR International Pty Ltd. NVivo. https://www.qsrinternational.com/nvivo-qualitative-data-analysis-software/home. 2018.

[49] Lincoln YS, Guba EG. Lincoln and Guba’s evaluative criteria. Nat Inq. 1985.

[50] Schulz KF, Altman DG, Moher D, CONSORT Group. CONSORT 2010 statement: updated guidelines for reporting parallel group randomised trials. BMJ. 2010;340:c332. doi: 10.1136/bmj.c332 20332509 PMC2844940

[51] McClure NS, Sayah FA, Ohinmaa A, Johnson JA. Minimally Important Difference of the EQ-5D-5L Index Score in Adults with Type 2 Diabetes. Value Health. 2018;21(9):1090–7. doi: 10.1016/j.jval.2018.02.007 30224114

[52] McClure NS, Sayah FA, Xie F, Luo N, Johnson JA. Instrument-Defined Estimates of the Minimally Important Difference for EQ-5D-5L Index Scores. Value Health. 2017;20(4):644–50. doi: 10.1016/j.jval.2016.11.015 28408007

[53] Coretti S, Ruggeri M, McNamee P. The minimum clinically important difference for EQ-5D index: a critical review. Expert Rev Pharmacoecon Outcomes Res. 2014;14(2):221–33. doi: 10.1586/14737167.2014.894462 24625040

[54] Nolan CM, Longworth L, Lord J, Canavan JL, Jones SE, Kon SSC, et al. The EQ-5D-5L health status questionnaire in COPD: validity, responsiveness and minimum important difference. Thorax. 2016;71(6):493–500. doi: 10.1136/thoraxjnl-2015-207782 27030578 PMC4893131

[55] Luo N, Johnson J, Coons SJ. Using instrument-defined health state transitions to estimate minimally important differences for four preference-based health-related quality of life instruments. Med Care. 2010;48(4):365–71. doi: 10.1097/mlr.0b013e3181c162a2 20355266

[56] Meyerowitz-Katz G, Ravi S, Arnolda L, Feng X, Maberly G, Astell-Burt T. Rates of Attrition and Dropout in App-Based Interventions for Chronic Disease: Systematic Review and Meta-Analysis. J Med Internet Res. 2020;22(9):e20283. doi: 10.2196/20283 32990635 PMC7556375

[57] Meyer VM, Benjamens S, Moumni ME, Lange JFM, Pol RA. Global Overview of Response Rates in Patient and Health Care Professional Surveys in Surgery: A Systematic Review. Ann Surg. 2022;275(1):e75–81. doi: 10.1097/SLA.0000000000004078 32649458 PMC8683255

[58] Hendra R, Hill A. Rethinking Response Rates: New Evidence of Little Relationship Between Survey Response Rates and Nonresponse Bias. Eval Rev. 2019;43(5):307–30. doi: 10.1177/0193841X18807719 30580577

[59] Gillies K, Kearney A, Keenan C, Treweek S, Hudson J, Brueton VC, et al. Strategies to improve retention in randomised trials. Cochrane Database Syst Rev. 2021;3(3):MR000032. doi: 10.1002/14651858.MR000032.pub3 33675536 PMC8092429

[60] Laiteerapong N, Cooper JM, Skandari MR, Clarke PM, Winn AN, Naylor RN, et al. Individualized Glycemic Control for U.S. Adults With Type 2 Diabetes: A Cost-Effectiveness Analysis. Ann Intern Med. 2018;168(3):170–8. doi: 10.7326/M17-0537 29230472 PMC5989575

[61] Fisher L, Mullan JT, Arean P, Glasgow RE, Hessler D, Masharani U. Diabetes distress but not clinical depression or depressive symptoms is associated with glycemic control in both cross-sectional and longitudinal analyses. Diabetes Care. 2010;33(1):23–8. doi: 10.2337/dc09-1238 19837786 PMC2797978

[62] Beard E, Clark M, Hurel S, Cooke D. Do people with diabetes understand their clinical marker of long-term glycemic control (HbA1c levels) and does this predict diabetes self-care behaviours and HbA1c?. Patient Educ Couns. 2010;80(2):227–32. doi: 10.1016/j.pec.2009.11.008 20036098

[63] Lorig KR, Holman H. Self-management education: history, definition, outcomes, and mechanisms. Ann Behav Med. 2003;26(1):1–7. doi: 10.1207/S15324796ABM2601_01 12867348

[64] Unger WR, Buelow JM. Hybrid concept analysis of self-management in adults newly diagnosed with epilepsy. Epilepsy Behav. 2009;14(1):89–95. doi: 10.1016/j.yebeh.2008.09.002 18796337

[65] Adair JG. The Hawthorne Effect: A Reconsideration of the Methodological Artifact. J Appl Psychol. 1984;69:334–45.

[66] Office for Health Improvement & Disparities. Public health profiles. https://fingertips.phe.org.uk/ 2022 November 5.

[67] St Helens Borough Council. Local Insight. https://sthelens.communityinsight.org/ 2022 November 5.

[68] Capodaglio P, Castelnuovo G, Brunani A, Vismara L, Villa V, Capodaglio EM. Functional limitations and occupational issues in obesity: a review. Int J Occup Saf Ergon. 2010;16(4):507–23. doi: 10.1080/10803548.2010.11076863 21144269

[69] Westall SJ, Narayanan RP, Watmough S, Irving G, Furlong N, McNulty S, et al. The individualisation of glycaemic targets in response to patient characteristics in type 2 diabetes: a scoping review. Clin Med (Lond). 2022;22(3):257–65. doi: 10.7861/clinmed.2021-0764 35443970 PMC9135095

