# Supplementary material for: Feasibility of glycated haemoglobin target-setting in adults with diabetes: A mixed-methods study
Source: PLoS One. 2026 Jan 5;21(1):e0317162. doi: 10.1371/journal.pone.0317162 (PMC12768356; doi:10.1371/journal.pone.0317162)
Supplement: S3 File — (PDF) [file pone.0317162.s003.pdf]

Patient interview topic guide and protocol (Study B).
IRAS ID: **291254**

Venue: Via telephone

Please ensure a quiet and distraction-free environment for the interview. Study B interview dates and times should be arranged during patient visits to the diabetes centre during Study A final visit. Interviews should be carried after participants have completed study A.

Proposed duration: 15 – 30 minutes.

Structure: Call participant and inform them that the call is recorded for research purposes and that their data will remain confidential. Briefly summarise the purpose of the study. Ensure participant has read and understood Study B participant information sheet. Answer any questions they may have. Consent already obtained during Study A.

The interview will begin, (start audio recording):
(Topics in blue, underlined. Themes identified in **bold.** Probing questions indented)

Greet participant, introductions.

"Please tell me, how long have you had diabetes?" (*easy question/ice-breaker*)
    "How were you diagnosed/what happened?"

Perceptions on the use of 'A1c' targets

"**What do you know about the goals/targets for your 'A1c'**?"
    "What do these mean personally for you?"
    "Why do you think 'A1c' is important?"
"**How did you feel about being given an 'A1c' target in the study**?"
    "Did it make you feel driven to do better?"
    "Did it make you disheartened and demotivated?"
    "If you were doing well, did it make you relax your efforts towards your diabetes?"
    "Did it make you feel elated?"
    "if you weren't doing well, did it make you more motivated or less motivated?"
"Had you been given an 'A1c' target before the study?"
    "How did it make you feel?"
    "Why did it make you feel like this?"
"What is your opinion on using 'A1c' targets in people with diabetes?"

Individualisation of 'A1c' targets

"There is a lot of research that tells us that **people with diabetes should be given individualised goals for 'A1c'**. What are your views about this?"
    "Can you tell me more about that?"
    "In your opinion, what are the positive things about having an 'A1c' target?"
    "In your opinion, what are the negative things about having and 'A1c' target?"

Transcribe the interview as soon as possible after completion.

Patient interview topic guide and protocol (Study B).
IRAS ID: **291254**

Diabetes and mental health

"Do you feel that diabetes has had an effect on your sense of well-being?"

"In what way?"

"How have you dealt with this?"

"Do you think that diabetes has affected your mental health?"

"how/in what way?"

"why do you think that diabetes has had this effect?"

"What do you think would make you feel better about living with diabetes?"

Study Processes

"How did you feel about being involved in diabetes research?"

"People in this study, including you, were randomly put into different groups with different interventions. How do you feel about this?"

"If you don't mind, why?"

"If you do mind, why?"

"How did you feel about the time commitments for the study?"

"Did you find the leaflet about 'A1c' useful?"

"if so, in what way?"

"if not, why not?"

"We testing to see if we were able to keep enough people in the study. You stayed/did not stay in the study until the end. Why was this?"

"Is there anything that we should have done differently?"

"Did you find the information sheets and letters in the study easy to understand?"

"can you give further information on that?"

"how could we make the documents better?"

"There were a few questionnaires to complete during the study. How did you find these?"

"Would you take part in research again?"

Probing:
Active listening to guide further questioning
Appropriate use of silence
Politely interrupt and re-focus the interview if going off-topic
Clarify/elaborate/expand

Closing:
"Is there anything else you would like to add?"

Once complete, answer any questions the interviewee may have, stop the recording and thank the interviewee for their participation.

Transcribe the interview as soon as possible after completion.
