# Supplementary material for: Feasibility of glycated haemoglobin target-setting in adults with diabetes: A mixed-methods study
Source: PLoS One. 2026 Jan 5;21(1):e0317162. doi: 10.1371/journal.pone.0317162 (PMC12768356; doi:10.1371/journal.pone.0317162)
Supplement: S4 File — (PDF) [file pone.0317162.s004.pdf]

Edge Hill University

St Helens and Knowsley Teaching Hospitals NHS Trust

Healthcare professionals interview topic guide and protocol
(Study C). IRAS ID: **291254**

Venue: Via telephone/video call

Ensure a distraction-free environment is located for telephone/video calls. Ensure the interview has appropriate access to telephone/video call facilities within the trust. Study C interview dates and times should be arranged via email with the healthcare professional.

Proposed duration: 15 – 30 minutes.

Structure: Call participant and inform them that the call is recorded for research purposes and that their data will remain confidential. Briefly summarise the purpose of the study. Ensure participant has read and understood Study C participant information sheet. Answer any questions they may have. Ensure consent form has been completed.

The interview will begin, (start audio recording):
(Topics in blue, underlined. Themes identified in **bold.** Probing questions indented.)

Greet participant, introductions.

"Can you tell me a little bit about your professional background as a diabetes healthcare professional?" (easy question/ice-breaker)

Perceptions on the use of HbA1c targets

"HbA1c targets are universally used to aid management decisions in people with diabetes. We know it is beneficial to help patients achieve these targets. **What do you think patients' understanding of HbA1c is?"**
"What sort of discussions would you normally have with a patient on the topic of HbA1c?"
"What do you think patients' views on HbA1c targets are?"
    "What do you think could be done to help improve patient perceptions of HbA1c?"
"What are your views on the use of HbA1c targets in people with diabetes?"
"What are the main advantages and disadvantages to using HbA1c targets in the management of people with diabetes?"
"If you knew that HbA1c targets had an impact on the mental health of people with diabetes, in what way would it change your discussions with patients?"

Individualisation of HbA1c targets

A lot of research has suggested that HbA1c targets should be individualised to the patient.
"How has this informed your practice?"
"What approach do you take to individualising HbA1c targets?"
    "Have you found that this is beneficial?"
    "If so, in what way?"
    "If not, why not?"
"In what way would you say HbA1c target-setting in people with diabetes could be improved?"

Transcribe the interview as soon as possible after completion.

Healthcare professionals interview topic guide and protocol (Study C). IRAS ID: **291254**

Diabetes and mental health

Mental health comorbidities are known to have an impact upon the self-management abilities of people with diabetes. **"What do you think is the best way to improve the mental health of people with diabetes?"**

"Do you think setting HbA1c targets in people with diabetes has an impact upon their mental health?"

"How can healthcare professionals be supported to deliver better care for people with diabetes?"

Probing:

Active listening to guide further questioning
Appropriate use of silence
Politely interrupt and re-focus the interview if going off-topic
Clarify/elaborate/expand

Closing:

"Is there anything else you would like to add?"

Once complete, answer any questions the interviewee may have, stop the recording and thank the interviewee for their participation.

Transcribe the interview as soon as possible after completion.
