# Supplementary material for: Feasibility of glycated haemoglobin target-setting in adults with diabetes: A mixed-methods study
Source: PLoS One. 2026 Jan 5;21(1):e0317162. doi: 10.1371/journal.pone.0317162 (PMC12768356; doi:10.1371/journal.pone.0317162)
Supplement: Attachment — Submitted filename: Response to reviewers.docx [file pone.0317162.s006.docx]

21/2/25

**Re: PLOS ONE Decision: Revision required [PONE-D-24-59147] - [EMID:7fa3d61f3a31db3b]**

Dear Editor and Reviewers,

On behalf of my co-authors, I would like to thank you for reviewing our article entitled “Psychometric and biomedical outcomes of glycated haemoglobin target-setting in adults with type 1 and type 2 diabetes: Protocol for a mixed-methods parallel-group randomised feasibility study” with opportunity to revise and resubmit.

We found the reviewer’s comments helpful in revising the manuscript and have carefully considered their suggestions. We revised the manuscript in accordance with the reviewer’s comments and the journal requirements.

Below, we have outlined journal requirements and reviewer comments in black followed by our responses in red, prefaced “Author response”. Corresponding changes are highlighted in the tracked manuscript text file in red using the ‘track changes’ function.

Yours Sincerely,

Journal requirements **and** author responses

1. Please ensure that your manuscript meets PLOS ONE's style requirements, including those for file naming.

Author response:

We have reformatted the manuscript to comply with PLOS ONE’s style requirements. Changes are marked using the ‘tracked changes’ function.

2. We note that you have provided funding information that is not currently declared in your Funding Statement. However, funding information should not appear in the Acknowledgments section or other areas of your manuscript. We will only publish funding information present in the Funding Statement section of the online submission form.

Please remove any funding-related text from the manuscript and let us know how you would like to update your Funding Statement.

Author response:

Apologies for the confusion. The authors received no specific grants to undertake this research. We would like to update the following sections:

Funding statement: “This work forms part of the first authors PhD. S.J.W. is supported by the Department of Diabetes and Endocrinology, St Helens and Knowsley Teaching Hospitals NHS Trust, UK.” (Acknowledgements removed from the manuscript using the ‘tracked changes’ function)

Financial disclosure/funding information: “The authors received no specific funding for this work.”

3. We note that this data set consists of interview transcripts. Can you please confirm that all participants gave consent for interview transcript to be published?

Consent for interviews included consent for anonymised interview transcript publication. We have included a sentence in the manuscript to confirm this.

If they DID provide consent for these transcripts to be published, please also confirm that the transcripts do not contain any potentially identifying information (or let us know if the participants consented to having their personal details published and made publicly available). We consider the following details to be identifying information:

- Names, nicknames, and initials

- Age more specific than round numbers

- GPS coordinates, physical addresses, IP addresses, email addresses

- Information in small sample sizes (e.g. 40 students from X class in X year at X university)

- Specific dates (e.g. visit dates, interview dates)

- ID numbers

We confirm that there is no identifiable information in the manuscript, including the transcript quotes.

Reviewers' comments **and** author responses:

1. Is the manuscript technically sound, and do the data support the conclusions?

The manuscript must describe a technically sound piece of scientific research with data that supports the conclusions. Experiments must have been conducted rigorously, with appropriate controls, replication, and sample sizes. The conclusions must be drawn appropriately based on the data presented.

Reviewer #1: Yes

2. Has the statistical analysis been performed appropriately and rigorously?

Reviewer #1: Yes

3. Have the authors made all data underlying the findings in their manuscript fully available?

The [PLOS Data policy](http://track.editorialmanager.com/CL0/http:%2F%2Fwww.plosone.org%2Fstatic%2Fpolicies.action%23sharing/1/010f0194b845a0db-544c71ef-d03c-4898-85b4-ae07ed552947-000000/8UfsVZePqmPIsciIBbfr1SG7Iu1OXxes4aCySuyCYfI=195) requires authors to make all data underlying the findings described in their manuscript fully available without restriction, with rare exception (please refer to the Data Availability Statement in the manuscript PDF file). The data should be provided as part of the manuscript or its supporting information, or deposited to a public repository. For example, in addition to summary statistics, the data points behind means, medians and variance measures should be available. If there are restrictions on publicly sharing data—e.g. participant privacy or use of data from a third party—those must be specified.

Reviewer #1: Yes

4. Is the manuscript presented in an intelligible fashion and written in standard English?

PLOS ONE does not copyedit accepted manuscripts, so the language in submitted articles must be clear, correct, and unambiguous. Any typographical or grammatical errors should be corrected at revision, so please note any specific errors here.

Reviewer #1: Yes

5. Review Comments to the Author

Please use the space provided to explain your answers to the questions above. You may also include additional comments for the author, including concerns about dual publication, research ethics, or publication ethics. (Please upload your review as an attachment if it exceeds 20,000 characters)

**Reviewer #1:** The authors conduct a feasibility study with a randomized, mixed-methods study design to evaluate the impact of setting explicit HbA1c targets in adults with diabetes. They recruited fifty participants randomly assigned to two arms with 1:1 ratio, one targeting 5 mmol/mol above and another below current HbA1c. the results showed no significant between-group differences in self-reported measures or HbA1c levels. But significant results were observed for levels of distress, self-efficacy, and subsequent HbA1c readings. Some specific motivators and demotivators to reach glycaemic targets were observed through interview.

1.Line 215. Outliers were noted and removed prior to analysis. Please provide the information how you determine the outlier and how many were excluded

This detail has now been included.

2.Table 3. It seems that many characters are different between groups (A vs B or completers vs non-completers). It would be good to provide the p-value evaluating the difference between group A and group B and another for evaluating the difference between completers and non-completers.

Table 3 updated.

3.Based on Table 3, some sample characteristics differed between group assignments. Were these covariates adjusted in the downstream analysis? Without adjustments, the results would be due to the confounding.

Multiple linear regression analysis was conducted to identify confounding factors (gender, age, diabetes duration, IMD decile, BMI) potentially affecting outcomes. This was commented upon in the results (subheading: confounders) and discussion (subheading: strengths and limitations). Now also added in methods – data analysis.

4.Based on Table 3, the sample characteristics difference between completers and non-completers brings the concern on the validity and generalizability of the final results and conclusion.

Now commented upon in strengths and limitations.

5.The current version of manuscript is way too lengthy with around 100 pages. Some work should be made.

Noted. Article length considerably shortened. (word count 21573 -> 13419)

6. PLOS authors have the option to publish the peer review history of their article ([what does this mean?](https://track.editorialmanager.com/CL0/https:%2F%2Fjournals.plos.org%2Fplosone%2Fs%2Feditorial-and-peer-review-process%23loc-peer-review-history/1/010f0194b845a0db-544c71ef-d03c-4898-85b4-ae07ed552947-000000/cdJiy7pbpAthmHJrkagha0ZShELwdRwOeDcCfW8NoXs=195)). If published, this will include your full peer review and any attached files.


If you choose “no”, your identity will remain anonymous but your review may still be made public.


Do you want your identity to be public for this peer review? For information about this choice, including consent withdrawal, please see our [Privacy Policy](https://track.editorialmanager.com/CL0/https:%2F%2Fwww.plos.org%2Fprivacy-policy/1/010f0194b845a0db-544c71ef-d03c-4898-85b4-ae07ed552947-000000/yS1iU1MJJF9mm-kI-F2cGsZK6hQF1xE5yyfPu5SqDp8=195).

Reviewer #1: No

------END OF RESPONSE------
