# Supplementary material for: Feasibility of glycated haemoglobin target-setting in adults with diabetes: A mixed-methods study
Source: PLoS One. 2026 Jan 5;21(1):e0317162. doi: 10.1371/journal.pone.0317162 (PMC12768356; doi:10.1371/journal.pone.0317162)
Supplement: Attachment — Submitted filename: Response to reviewers 1125.docx [file pone.0317162.s007.docx]

14/11/25

**Re: PLOS ONE Decision: Revision required [PONE-D-24-59147] - [EMID:7fa3d61f3a31db3b]**

Dear Editor and Reviewers,

On behalf of my co-authors, I would like to thank you for reviewing our article entitled “Feasibility of glycated haemoglobin target-setting in adults with diabetes: a mixed-methods study” with opportunity to revise and resubmit.

We found the reviewer’s comments helpful in revising the manuscript and have carefully considered their suggestions. We revised the manuscript in accordance with the reviewer’s comments and the journal requirements.

Below, we have outlined journal requirements and reviewer comments in black followed by our responses in red, prefaced “Author response”. Corresponding changes are highlighted in the tracked manuscript text file in red using the ‘track changes’ function.

Yours Sincerely,

Dr Sam Westall

On Behalf of all the authors

Reviewers' comments **and** author responses:

1. If the authors have adequately addressed your comments raised in a previous round of review and you feel that this manuscript is now acceptable for publication, you may indicate that here to bypass the "Comments to the Author" section, enter your conflict of interest statement in the "Confidential to Editor" section, and submit your "Accept" recommendation.
   Reviewer #1: All comments have been addressed

Reviewer #2: All comments have been addressed

2. Is the manuscript technically sound, and do the data support the conclusions?
   The manuscript must describe a technically sound piece of scientific research with data that supports the conclusions.

Reviewer #1: (No response)

Reviewer #2: Partly

3. Has the statistical analysis been performed appropriately and rigorously?

Reviewer #1: (No response)

Reviewer #2: Yes

4. Have the authors made all data underlying the findings in their manuscript fully available?

The capital PLOS data policy requires authors to make all data underlying the findings described in their manuscript fully available without restriction, with rare exception (please refer to the data availability statement in the manuscript PDF file). The data should be provided as part of the manuscript or its supporting information, or deposited to a public repository. For example, in addition to summary statistics, the data points behind means, medians and variance measures should be available. If there are restrictions on publicly sharing data- e.g. participant privacy or use of data from a third party- those must be specified.

Reviewer #1: (no response)

Reviewer #2: Yes

5. Is the manuscript presented in an intelligible fashion and written in standard English?
   PLOS ONE does not copyedit accepted manuscripts, so the language in submitted articles must be clear, correct, and unambiguous. Any typographical or grammatical errors should be corrected at revision, so please note any specific errors here.

Reviewer #1: (No Response)

Reviewer #2: Yes

5. Review Comments to the Author

Please use the space provided to explain your answers to the questions above. You may also include additional comments for the author, including concerns about dual publication, research ethics, or publication ethics. (Please upload your review as an attachment if it exceeds 20,000 characters)

Reviewer #1: (No Response)

Reviewer #2: I am writing to inform you that I do not recall whether I have previously evaluated this manuscript or if I have been asked to provide a third opinion. Nevertheless, I have carefully read through the manuscript and have provided my comments for your consideration.

Title: The title is long and dense, making it harder to quickly digest.

Title amended.

Abstract: After reading the abstract, I found it somewhat lengthy and noticed that it lacks key methodological details, particularly regarding the analysis of both qualitative and quantitative data.

Abstract shortened and key methodological details included.

Background: The background effectively covers a wide range of relevant topics, including the clinical importance of glycated haemoglobin (HbA1c), the rationale for individualized targets, associated comorbidities, and healthcare system challenges. However, the section is too lengthy and somewhat repetitive, particularly in explaining the complications associated with poor glycaemic control. For instance, the negative health outcomes are listed extensively, which could be condensed for impact. In addition, the structure could be more cohesive. It jumps between patient-level challenges, healthcare provider challenges, treatment outcomes, and statistics. Grouping similar ideas together and using clearer transitions would help the reader follow the argument more easily.

Background shortened and improved for better flow.

Aim and Objectives: The aims and objectives are generally clear and well-structured, reflecting the exploratory nature of the study and its focus on both clinical and psychosocial outcomes. However, the phrasing of the aim could be clearer—terms like "relaxed or intensified glycated haemoglobin targets" may be ambiguous to some readers and would benefit from clarification. The use of Roman numerals to list sub-points under the aim is somewhat confusing and unnecessary, especially as these points are already covered in the objectives. Additionally, there is some redundancy between the aim and Objective I, both of which address feasibility aspects. Objective 2 is overly broad, combining multiple outcome variables that could be better organized into psychosocial and clinical categories. Terminology such as "acceptability" and "experiences, views and opinions" should be more precise, and the inconsistent use of past and present tense between the aim and objectives could be revised for consistency. Overall, while the content is appropriate, the section would benefit from clearer wording, streamlined structure, and greater precision in describing the study's intent and scope.

Clarity improved. Redundancy removed. Precision of language improved. Tense consistency amended.

Participants: The participants section provides a basic overview of inclusion criteria and recruitment procedures but falls short in terms of methodological depth and clarity. While it is appropriate to define the target population (adults with type 1 or type 2 diabetes and specified HbA1c range), referring readers to an external protocol for exclusion criteria is not ideal; at least a summary of key exclusions should be included for transparency.

Exclusion criteria now included, rather than referring the reader to the published protocol.

Recruitment: The recruitment strategy—via mailed invitations prior to clinic visits—is briefly described, but lacks Detail on how many participatory patients were approached, how many agreed to participate, and whether there were any notable patterns in non-response or attrition. The mention of face and content validity review by a service user group is a strength, yet the process and its impact on document revisions are not elaborated. Furthermore, the sampling approach for the qualitative component is only superficially described.

This detail (number screened, eligible, enrolled, excluded, etc) is included in the Figure 1 CONSORT flow chart, now referenced in this section.

Service user group feedback and process details included.

Further detail provided on the qualitative component sampling approach.

Data Analysis: The data analysis section demonstrates a generally sound approach to handling quantitative data in a feasibility study, with appropriate attention given to descriptive statistics, data normality, and the use of inferential tests to explore trends. However, several issues limit its clarity and methodological robustness. Firstly, repeatedly referring readers to a previously published protocol diminishes the transparency of this standalone report; Key components- especially analytic decisions- should be more fully explained within the current manuscript. While the use of both parametric (t-test) and non-parametric (Wilcoxon, Mann-Whitney U) tests is appropriate based on distribution, the rationale for using inferential statistics in a feasibility study- despite acknowledging underpowered sample size- remains questionable and should be so it could be misleading if interpreted as evidence of effectiveness. More emphasis should be placed on interpreting these results cautiously. The qualitative data analysis section reflects a generally rigorous and methodologically sound approach, particularly for a feasibility study aiming to explore patient and professional perspectives. The use of the framework method of thematic analysis is appropriate for studies that involve multiple researchers and predefined areas of interest, and the integration of Nvivo software adds a layer of transparency and auditability. The strategy of combining deductive (predetermined themes) with inductive (emergent themes) coding demonstrates a balanced approach that allows for both structured inquiry and responsiveness to participant voices.

Additional detail has been added instead of referring the reader to the protocol.

Additional information provided on analytical processes and decisions.

Further emphasis on the importance of interpreting results with caution added.

Results: The results section is clear, transparent, and responsive to reviewer concerns, with appropriate statistical analysis and a balanced presentation of findings. However, the small sample size, high dropout rate, and lack and some lack of detail (especially in qualitative reporting and integration) limit the strength and generalizability of the conclusions. For feasibility study, these are not fatal flaws, but they do highlight important considerations for future research.

Additional detail provided to qualitative results. Commentary on data integration inserted in discussion, including how method triangulation, etc, enhanced credibility of findings.

Discussion: This discussion is rich in data interpretation and insight and demonstrates good scholarly rigor. To enhance clarity and impact, consider restructuring for flow, removing redundancies, expanding on key unexpected findings, explicitly linking qualitative and quantitative data, and adding a limitations section. These refinements strengthen the narrative and make the implications more accessible for readers and future researchers.

Discussion shortened, restructures, and now demonstrate greater caution with result interpretation. Explicit linkages to quantitative and qualitative findings elaborated upon.

Strength and limitations: The study's strengths include a clear focus on feasibility with transparent acknowledgment of its limited statistical power, alongside thorough demographic reporting that allows assessment of generalizability within a defined local context. The use of a mixed-methods design and multiple forms of triangulation enhances the credibility and trustworthiness of the findings, demonstrating methodological rigor. However, significant limitations arise from recruitment at a single centre with a predominantly white population and higher local diabetes prevalence, which limits broader applicability of the results. The small sample size and wide confidence intervals reflect the underpowered nature of the study, restricting meaningful statistical conclusions. Additionally, attrition bias is a concern, particularly due to the higher dropout rates among younger participants whose reasons for withdrawal were often unreported. These factors weaken the internal validity and highlight the need for multicentre recruitment and retention strategies in future research to improve representativeness and reduce bias.

Agreed and all discussed within strengths and limitations

Conclusion: The conclusion effectively highlights the study's contribution to the limited evidence on psychometric outcomes in diabetes and the potential benefits of individualized glycated haemoglobin targets. It appropriately emphasizes the feasibility of a larger, more definitive trial and reinforces the importance of shared decision-making In clinical practice. However, the conclusion tends to overstate the preliminary findings by suggesting improvements in patient-reported outcomes (PROs) and biomedical measures without sufficiently acknowledging the study's limited statistical power and potential biases discussed earlier. While it references existing evidence for individualized targets, it could better contextualize how this study adds novel insights, particularly regarding PROs and patient experience. The discussion of the complex relationship between psychological and physical health is valuable but somewhat broad and would benefit from clearer linkage to the study's specific findings. Overall, the conclusion is optimistic but would gain from a more cautious tone that clearly differentiates preliminary feasibility results from conclusive evidence.

Flow improved. Conclusion has been adapted to adopt a more cautious interpretation of findings, acknowledging the limitations of the study. Improved contextualisation of the novel insights provided by the study.

6. PLOS authors have the option to publish the peer review history of their article ([what does this mean?](https://track.editorialmanager.com/CL0/https:%2F%2Fjournals.plos.org%2Fplosone%2Fs%2Feditorial-and-peer-review-process%23loc-peer-review-history/1/010f0194b845a0db-544c71ef-d03c-4898-85b4-ae07ed552947-000000/cdJiy7pbpAthmHJrkagha0ZShELwdRwOeDcCfW8NoXs=195)). If published, this will include your full peer review and any attached files.


If you choose “no”, your identity will remain anonymous but your review may still be made public.


Do you want your identity to be public for this peer review? For information about this choice, including consent withdrawal, please see our [Privacy Policy](https://track.editorialmanager.com/CL0/https:%2F%2Fwww.plos.org%2Fprivacy-policy/1/010f0194b845a0db-544c71ef-d03c-4898-85b4-ae07ed552947-000000/yS1iU1MJJF9mm-kI-F2cGsZK6hQF1xE5yyfPu5SqDp8=195).

Reviewer #1: No

Reviewer #2: No

------END OF RESPONSE------
