# Supplementary material for: Feasibility of glycated haemoglobin target-setting in adults with diabetes: A mixed-methods study
Source: PLoS One. 2026 Jan 5;21(1):e0317162. doi: 10.1371/journal.pone.0317162 (PMC12768356; doi:10.1371/journal.pone.0317162)
Supplement: Peer review history

# Peer review: Decision Letter 0

**DOI:** 10.1371/journal.pone.0317162.r001

**Authors:** Yee Gary Ang

30 Jan 2025

Dear Dr. Westall,

Thank you for submitting your manuscript to PLOS ONE. After careful consideration, we feel that it has merit but does not fully meet PLOS ONE’s publication criteria as it currently stands. Therefore, we invite you to submit a revised version of the manuscript that addresses the points raised during the review process.

**We have invited several reviewers but only one responded with major revision. Please review and consider resubmitting.**

Please submit your revised manuscript by Mar 16 2025 11:59PM. If you will need more time than this to complete your revisions, please reply to this message or contact the journal office at plosone@plos.org . When you're ready to submit your revision, log on to https://www.editorialmanager.com/pone/ and select the 'Submissions Needing Revision' folder to locate your manuscript file.

- A rebuttal letter that responds to each point raised by the academic editor and reviewer(s). You should upload this letter as a separate file labeled 'Response to Reviewers'.
- A marked-up copy of your manuscript that highlights changes made to the original version. You should upload this as a separate file labeled 'Revised Manuscript with Track Changes'.
- An unmarked version of your revised paper without tracked changes. You should upload this as a separate file labeled 'Manuscript'.

If you would like to make changes to your financial disclosure, please include your updated statement in your cover letter. Guidelines for resubmitting your figure files are available below the reviewer comments at the end of this letter.

If applicable, we recommend that you deposit your laboratory protocols in protocols.io to enhance the reproducibility of your results. Protocols.io assigns your protocol its own identifier (DOI) so that it can be cited independently in the future. For instructions see: https://journals.plos.org/plosone/s/submission-guidelines#loc-laboratory-protocols . Additionally, PLOS ONE offers an option for publishing peer-reviewed Lab Protocol articles, which describe protocols hosted on protocols.io. Read more information on sharing protocols at https://plos.org/protocols?utm_medium=editorial-email&utm_source=authorletters&utm_campaign=protocols .

We look forward to receiving your revised manuscript.

Kind regards,

Yee Gary Ang, MBBS MPH

Academic Editor

PLOS ONE

**Journal Requirements:**

1. When submitting your revision, we need you to address these additional requirements.

Please ensure that your manuscript meets PLOS ONE's style requirements, including those for file naming. The PLOS ONE style templates can be found at 

https://journals.plos.org/plosone/s/file?id=wjVg/PLOSOne_formatting_sample_main_body.pdf and 

https://journals.plos.org/plosone/s/file?id=ba62/PLOSOne_formatting_sample_title_authors_affiliations.pdf

2. Thank you for stating the following in the Acknowledgments Section of your manuscript: 

This work forms part of the first author’s PhD. S.J.W. is supported by the Department of Diabetes and Endocrinology, St Helens and Knowsley Teaching Hospitals NHS Trust, UK. 

We note that you have provided funding information that is not currently declared in your Funding Statement. However, funding information should not appear in the Acknowledgments section or other areas of your manuscript. We will only publish funding information present in the Funding Statement section of the online submission form. 

Please remove any funding-related text from the manuscript and let us know how you would like to update your Funding Statement. Currently, your Funding Statement reads as follows: 

The author(s) received no specific funding for this work.

Please include your amended statements within your cover letter; we will change the online submission form on your behalf.

3. We note that this data set consists of interview transcripts. Can you please confirm that all participants gave consent for interview transcript to be published?

If they DID provide consent for these transcripts to be published, please also confirm that the transcripts do not contain any potentially identifying information (or let us know if the participants consented to having their personal details published and made publicly available). We consider the following details to be identifying information:

- Names, nicknames, and initials

- Age more specific than round numbers

- GPS coordinates, physical addresses, IP addresses, email addresses

- Information in small sample sizes (e.g. 40 students from X class in X year at X university)

- Specific dates (e.g. visit dates, interview dates)

- ID numbers

Or, if the participants DID NOT provide consent for these transcripts to be published:

- Provide a de-identified version of the data or excerpts of interview responses

- Provide information regarding how these transcripts can be accessed by researchers who meet the criteria for access to confidential data, including:

a) the grounds for restriction

b) the name of the ethics committee, Institutional Review Board, or third-party organization that is imposing sharing restrictions on the data

c) a non-author, institutional point of contact that is able to field data access queries, in the interest of maintaining long-term data accessibility.

d) Any relevant data set names, URLs, DOIs, etc. that an independent researcher would need in order to request your minimal data set.

For further information on sharing data that contains sensitive participant information, please see: https://journals.plos.org/plosone/s/data-availability#loc-human-research-participant-data-and-other-sensitive-data

If there are ethical, legal, or third-party restrictions upon your dataset, you must provide all of the following details (https://journals.plos.org/plosone/s/data-availability#loc-acceptable-data-access-restrictions):

a) A complete description of the dataset

b) The nature of the restrictions upon the data (ethical, legal, or owned by a third party) and the reasoning behind them

c) The full name of the body imposing the restrictions upon your dataset (ethics committee, institution, data access committee, etc)

d) If the data are owned by a third party, confirmation of whether the authors received any special privileges in accessing the data that other researchers would not have

e) Direct, non-author contact information (preferably email) for the body imposing the restrictions upon the data, to which data access requests can be sent

[Note: HTML markup is below. Please do not edit.]

Reviewers' comments:

Reviewer's Responses to Questions

Comments to the Author

1. Is the manuscript technically sound, and do the data support the conclusions?

The manuscript must describe a technically sound piece of scientific research with data that supports the conclusions. Experiments must have been conducted rigorously, with appropriate controls, replication, and sample sizes. The conclusions must be drawn appropriately based on the data presented.

Reviewer #1: Yes

**********

2. Has the statistical analysis been performed appropriately and rigorously?

Reviewer #1: Yes

**********

3. Have the authors made all data underlying the findings in their manuscript fully available?

The PLOS Data policy requires authors to make all data underlying the findings described in their manuscript fully available without restriction, with rare exception (please refer to the Data Availability Statement in the manuscript PDF file). The data should be provided as part of the manuscript or its supporting information, or deposited to a public repository. For example, in addition to summary statistics, the data points behind means, medians and variance measures should be available. If there are restrictions on publicly sharing data—e.g. participant privacy or use of data from a third party—those must be specified.

Reviewer #1: Yes

**********

4. Is the manuscript presented in an intelligible fashion and written in standard English?

PLOS ONE does not copyedit accepted manuscripts, so the language in submitted articles must be clear, correct, and unambiguous. Any typographical or grammatical errors should be corrected at revision, so please note any specific errors here.

Reviewer #1: Yes

**********

5. Review Comments to the Author

Please use the space provided to explain your answers to the questions above. You may also include additional comments for the author, including concerns about dual publication, research ethics, or publication ethics. (Please upload your review as an attachment if it exceeds 20,000 characters)

**Reviewer #1: ** The authors conduct a feasibility study with a randomized, mixed-methods study design to evaluate the impact of setting explicit HbA1c targets in adults with diabetes. They recruited fifty participants randomly assigned to two arms with 1:1 ratio, one targeting 5 mmol/mol above and another below current HbA1c. the results showed no significant between-group differences in self-reported measures or HbA1c levels. But significant results were observed for levels of distress, self-efficacy, and subsequent HbA1c readings. Some specific motivators and demotivators to reach glycaemic targets were observed through interview.

1.Line 215. Outliers were noted and removed prior to analysis. Please provide the information how you determine the outlier and how many were excluded

2.Table 3. It seems that many characters are different between groups (A vs B or completers vs non-completers). It would be good to provide the p-value evaluating the difference between group A and group B and another for evaluating the difference between completers and non-completers.

3.Based on Table 3, some sample characteristics differed between group assignments. Were these covariates adjusted in the downstream analysis? Without adjustments, the results would be due to the confounding.

4.Based on Table 3, the sample characteristics difference between completers and non-completers brings the concern on the validity and generalizability of the final results and conclusion.

5.The current version of manuscript is way too lengthy with around 100 pages. Some work should be made.

**********

6. PLOS authors have the option to publish the peer review history of their article (what does this mean? ). If published, this will include your full peer review and any attached files.

If you choose “no”, your identity will remain anonymous but your review may still be made public.

Do you want your identity to be public for this peer review? For information about this choice, including consent withdrawal, please see our Privacy Policy .

Reviewer #1: No

**********

[NOTE: If reviewer comments were submitted as an attachment file, they will be attached to this email and accessible via the submission site. Please log into your account, locate the manuscript record, and check for the action link "View Attachments". If this link does not appear, there are no attachment files.]

While revising your submission, please upload your figure files to the Preflight Analysis and Conversion Engine (PACE) digital diagnostic tool, https://pacev2.apexcovantage.com/ . PACE helps ensure that figures meet PLOS requirements. To use PACE, you must first register as a user. Registration is free. Then, login and navigate to the UPLOAD tab, where you will find detailed instructions on how to use the tool. If you encounter any issues or have any questions when using PACE, please email PLOS at figures@plos.org . Please note that Supporting Information files do not need this step.

# Author comment: Author response to Decision Letter 1

**DOI:** 10.1371/journal.pone.0317162.r002

24 Apr 2025

21/2/25

Re: PLOS ONE Decision: Revision required [PONE-D-24-59147] - [EMID:7fa3d61f3a31db3b]

Dear Editor and Reviewers,

On behalf of my co-authors, I would like to thank you for reviewing our article entitled “Psychometric and biomedical outcomes of glycated haemoglobin target-setting in adults with type 1 and type 2 diabetes: Protocol for a mixed-methods parallel-group randomised feasibility study” with opportunity to revise and resubmit.

We found the reviewer’s comments helpful in revising the manuscript and have carefully considered their suggestions. We revised the manuscript in accordance with the reviewer’s comments and the journal requirements.

Below, we have outlined journal requirements and reviewer comments in black followed by our responses in red, prefaced “Author response”. Corresponding changes are highlighted in the tracked manuscript text file in red using the ‘track changes’ function.

Yours Sincerely,

Journal requirements and author responses

1. Please ensure that your manuscript meets PLOS ONE's style requirements, including those for file naming.

Author response:

We have reformatted the manuscript to comply with PLOS ONE’s style requirements. Changes are marked using the ‘tracked changes’ function.

2. We note that you have provided funding information that is not currently declared in your Funding Statement. However, funding information should not appear in the Acknowledgments section or other areas of your manuscript. We will only publish funding information present in the Funding Statement section of the online submission form.

Please remove any funding-related text from the manuscript and let us know how you would like to update your Funding Statement.

Author response:

Apologies for the confusion. The authors received no specific grants to undertake this research. We would like to update the following sections:

Funding statement: “This work forms part of the first authors PhD. S.J.W. is supported by the Department of Diabetes and Endocrinology, St Helens and Knowsley Teaching Hospitals NHS Trust, UK.” (Acknowledgements removed from the manuscript using the ‘tracked changes’ function)

Financial disclosure/funding information: “The authors received no specific funding for this work.”

3. We note that this data set consists of interview transcripts. Can you please confirm that all participants gave consent for interview transcript to be published?

Consent for interviews included consent for anonymised interview transcript publication. We have included a sentence in the manuscript to confirm this.

If they DID provide consent for these transcripts to be published, please also confirm that the transcripts do not contain any potentially identifying information (or let us know if the participants consented to having their personal details published and made publicly available). We consider the following details to be identifying information:

- Names, nicknames, and initials

- Age more specific than round numbers

- GPS coordinates, physical addresses, IP addresses, email addresses

- Information in small sample sizes (e.g. 40 students from X class in X year at X university)

- Specific dates (e.g. visit dates, interview dates)

- ID numbers

We confirm that there is no identifiable information in the manuscript, including the transcript quotes.

Reviewers' comments and author responses:

1. Is the manuscript technically sound, and do the data support the conclusions?

The manuscript must describe a technically sound piece of scientific research with data that supports the conclusions. Experiments must have been conducted rigorously, with appropriate controls, replication, and sample sizes. The conclusions must be drawn appropriately based on the data presented.

Reviewer #1: Yes

________________________________________

2. Has the statistical analysis been performed appropriately and rigorously?

Reviewer #1: Yes

________________________________________

3. Have the authors made all data underlying the findings in their manuscript fully available?

The PLOS Data policy requires authors to make all data underlying the findings described in their manuscript fully available without restriction, with rare exception (please refer to the Data Availability Statement in the manuscript PDF file). The data should be provided as part of the manuscript or its supporting information, or deposited to a public repository. For example, in addition to summary statistics, the data points behind means, medians and variance measures should be available. If there are restrictions on publicly sharing data—e.g. participant privacy or use of data from a third party—those must be specified.

Reviewer #1: Yes

________________________________________

4. Is the manuscript presented in an intelligible fashion and written in standard English?

PLOS ONE does not copyedit accepted manuscripts, so the language in submitted articles must be clear, correct, and unambiguous. Any typographical or grammatical errors should be corrected at revision, so please note any specific errors here.

Reviewer #1: Yes

________________________________________

5. Review Comments to the Author

Please use the space provided to explain your answers to the questions above. You may also include additional comments for the author, including concerns about dual publication, research ethics, or publication ethics. (Please upload your review as an attachment if it exceeds 20,000 characters)

Reviewer #1: The authors conduct a feasibility study with a randomized, mixed-methods study design to evaluate the impact of setting explicit HbA1c targets in adults with diabetes. They recruited fifty participants randomly assigned to two arms with 1:1 ratio, one targeting 5 mmol/mol above and another below current HbA1c. the results showed no significant between-group differences in self-reported measures or HbA1c levels. But significant results were observed for levels of distress, self-efficacy, and subsequent HbA1c readings. Some specific motivators and demotivators to reach glycaemic targets were observed through interview.

1.Line 215. Outliers were noted and removed prior to analysis. Please provide the information how you determine the outlier and how many were excluded

This detail has now been included.

2.Table 3. It seems that many characters are different between groups (A vs B or completers vs non-completers). It would be good to provide the p-value evaluating the difference between group A and group B and another for evaluating the difference between completers and non-completers.

Table 3 updated.

3.Based on Table 3, some sample characteristics differed between group assignments. Were these covariates adjusted in the downstream analysis? Without adjustments, the results would be due to the confounding.

Multiple linear regression analysis was conducted to identify confounding factors (gender, age, diabetes duration, IMD decile, BMI) potentially affecting outcomes. This was commented upon in the results (subheading: confounders) and discussion (subheading: strengths and limitations). Now also added in methods – data analysis.

4.Based on Table 3, the sample characteristics difference between completers and non-completers brings the concern on the validity and generalizability of the final results and conclusion.

Now commented upon in strengths and limitations.

5.The current version of manuscript is way too lengthy with around 100 pages. Some work should be made.

Noted. Article length considerably shortened. (word count 21573 -> 13419)

________________________________________

6. PLOS authors have the option to publish the peer review history of their article (what does this mean?). If published, this will include your full peer review and any attached files.

If you choose “no”, your identity will remain anonymous but your review may still be made public.

Do you want your identity to be public for this peer review? For information about this choice, including consent withdrawal, please see our Privacy Policy.

Reviewer #1: No

------END OF RESPONSE------

Attachment Submitted filename: Response to reviewers.docx

# Peer review: Decision Letter 1

**DOI:** 10.1371/journal.pone.0317162.r003

**Authors:** Yee Gary Ang

4 Jun 2025

Dear Dr. Westall,

Thank you for submitting your manuscript to PLOS ONE. After careful consideration, we feel that it has merit but does not fully meet PLOS ONE’s publication criteria as it currently stands. Therefore, we invite you to submit a revised version of the manuscript that addresses the points raised during the review process.

==============================

**There were mixed reviews with one reviewer accepting and the other one recommended major revision**

==============================

Please submit your revised manuscript by Jul 19 2025 11:59PM. If you will need more time than this to complete your revisions, please reply to this message or contact the journal office at plosone@plos.org . When you're ready to submit your revision, log on to https://www.editorialmanager.com/pone/ and select the 'Submissions Needing Revision' folder to locate your manuscript file.

- A rebuttal letter that responds to each point raised by the academic editor and reviewer(s). You should upload this letter as a separate file labeled 'Response to Reviewers'.
- A marked-up copy of your manuscript that highlights changes made to the original version. You should upload this as a separate file labeled 'Revised Manuscript with Track Changes'.
- An unmarked version of your revised paper without tracked changes. You should upload this as a separate file labeled 'Manuscript'.

If you would like to make changes to your financial disclosure, please include your updated statement in your cover letter. Guidelines for resubmitting your figure files are available below the reviewer comments at the end of this letter.

If applicable, we recommend that you deposit your laboratory protocols in protocols.io to enhance the reproducibility of your results. Protocols.io assigns your protocol its own identifier (DOI) so that it can be cited independently in the future. For instructions see: https://journals.plos.org/plosone/s/submission-guidelines#loc-laboratory-protocols . Additionally, PLOS ONE offers an option for publishing peer-reviewed Lab Protocol articles, which describe protocols hosted on protocols.io. Read more information on sharing protocols at https://plos.org/protocols?utm_medium=editorial-email&utm_source=authorletters&utm_campaign=protocols .

We look forward to receiving your revised manuscript.

Kind regards,

Yee Gary Ang, MBBS MPH

Academic Editor

PLOS ONE

[Note: HTML markup is below. Please do not edit.]

Reviewers' comments:

Reviewer's Responses to Questions

**Comments to the Author**

Reviewer #1: All comments have been addressed

Reviewer #2: All comments have been addressed

**********

2. Is the manuscript technically sound, and do the data support the conclusions?

Reviewer #1: (No Response)

Reviewer #2: Partly

**********

3. Has the statistical analysis been performed appropriately and rigorously?

Reviewer #1: (No Response)

Reviewer #2: Yes

**********

4. Have the authors made all data underlying the findings in their manuscript fully available?

The PLOS Data policy

Reviewer #1: (No Response)

Reviewer #2: Yes

**********

5. Is the manuscript presented in an intelligible fashion and written in standard English?

Reviewer #1: (No Response)

Reviewer #2: Yes

**********

Reviewer #1: (No Response)

Reviewer #2: I am writing to inform you that I do not recall whether I have previously evaluated this manuscript or if I have been asked to provide a third opinion. Nevertheless, I have carefully read through the manuscript and have provided my comments for your consideration.

1) Title: The title is long and dense, making it harder to quickly digest.

2) Abstract: After reading the abstract, I found it somewhat lengthy and noticed that it lacks key methodological details, particularly regarding the analysis of both qualitative and quantitative data.

3) Background: The background effectively covers a wide range of relevant topics, including the clinical importance of glycated haemoglobin (HbA1c), the rationale for individualized targets, associated comorbidities, and healthcare system challenges. However, the section is too lengthy and somewhat repetitive, particularly in explaining the complications associated with poor glycaemic control. For instance, the negative health outcomes are listed extensively, which could be condensed for impact. In addition, the structure could be more cohesive. It jumps between patient-level challenges, healthcare provider challenges, treatment outcomes, and statistics. Grouping similar ideas together and using clearer transitions would help the reader follow the argument more easily.

4) Aim and Objectives: The aims and objectives are generally clear and well-structured, reflecting the exploratory nature of the study and its focus on both clinical and psychosocial outcomes. However, the phrasing of the aim could be clearer—terms like “relaxed or intensified glycated haemoglobin targets” may be ambiguous to some readers and would benefit from clarification. The use of Roman numerals to list sub-points under the aim is somewhat confusing and unnecessary, especially as these points are already covered in the objectives. Additionally, there is some redundancy between the aim and Objective 1, both of which address feasibility aspects. Objective 2 is overly broad, combining multiple outcome variables that could be better organized into psychosocial and clinical categories. Terminology such as “acceptability” and “experiences, views and opinions” should be more precise, and the inconsistent use of past and present tense between the aim and objectives could be revised for consistency. Overall, while the content is appropriate, the section would benefit from clearer wording, streamlined structure, and greater precision in describing the study’s intent and scope.

5) Participants: The participants section provides a basic overview of inclusion criteria and recruitment procedures but falls short in terms of methodological depth and clarity. While it is appropriate to define the target population (adults with type 1 or type 2 diabetes and specified HbA1c range), referring readers to an external protocol for exclusion criteria is not ideal; at least a summary of key exclusions should be included for transparency.

6) Recruitment: The recruitment strategy—via mailed invitations prior to clinic visits—is briefly described, but lacks detail on how many patients were approached, how many agreed to participate, and whether there were any notable patterns in non-response or attrition. The mention of face and content validity review by a service user group is a strength, yet the process and its impact on document revisions are not elaborated. Furthermore, the sampling approach for the qualitative component is only superficially described.

7) Data analysis: The Data Analysis section demonstrates a generally sound approach to handling quantitative data in a feasibility study, with appropriate attention given to descriptive statistics, data normality, and the use of inferential tests to explore trends. However, several issues limit its clarity and methodological robustness. Firstly, repeatedly referring readers to a previously published protocol diminishes the transparency of this standalone report; key components—especially analytic decisions—should be more fully explained within the current manuscript. While the use of both parametric (t-test) and non-parametric (Wilcoxon, Mann-Whitney U) tests is appropriate based on distribution, the rationale for using inferential statistics in a feasibility study—despite acknowledging underpowered sample size—remains questionable and could be misleading if interpreted as evidence of effectiveness. More emphasis should be placed on interpreting these results cautiously. The qualitative data analysis section reflects a generally rigorous and methodologically sound approach, particularly for a feasibility study aiming to explore patient and professional perspectives. The use of the Framework Method of thematic analysis is appropriate for studies that involve multiple researchers and predefined areas of interest, and the integration of NVivo software adds a layer of transparency and auditability. The strategy of combining deductive (pre-determined themes) with inductive (emergent themes) coding demonstrates a balanced approach that allows for both structured inquiry and responsiveness to participant voices.

8) Results: The Results section is clear, transparent, and responsive to reviewer concerns, with appropriate statistical analysis and a balanced presentation of findings. However, the small sample size, high dropout rate, and some lack of detail (especially in qualitative reporting and integration) limit the strength and generalizability of the conclusions. For a feasibility study, these are not fatal flaws, but they do highlight important considerations for future research.

9) Discussion: This discussion is rich in data interpretation and insight and demonstrates good scholarly rigor. To enhance clarity and impact, consider restructuring for flow, removing redundancies, expanding on key unexpected findings, explicitly linking qualitative and quantitative data, and adding a limitations section. These refinements will strengthen the narrative and make the implications more accessible for readers and future researchers.

10) Strength and limitations: The study’s strengths include a clear focus on feasibility with transparent acknowledgment of its limited statistical power, alongside thorough demographic reporting that allows assessment of generalizability within a defined local context. The use of a mixed-methods design and multiple forms of triangulation enhances the credibility and trustworthiness of the findings, demonstrating methodological rigor. However, significant limitations arise from recruitment at a single centre with a predominantly white population and higher local diabetes prevalence, which limits broader applicability of the results. The small sample size and wide confidence intervals reflect the underpowered nature of the study, restricting meaningful statistical conclusions. Additionally, attrition bias is a concern, particularly due to the higher dropout rates among younger participants whose reasons for withdrawal were often unreported. These factors weaken the internal validity and highlight the need for multicentre recruitment and retention strategies in future research to improve representativeness and reduce bias.

11) Conclusion: The conclusion effectively highlights the study’s contribution to the limited evidence on psychometric outcomes in diabetes and the potential benefits of individualized glycated haemoglobin targets. It appropriately emphasizes the feasibility of a larger, more definitive trial and reinforces the importance of shared decision-making in clinical practice. However, the conclusion tends to overstate the preliminary findings by suggesting improvements in patient-reported outcomes (PROs) and biomedical measures without sufficiently acknowledging the study’s limited statistical power and potential biases discussed earlier. While it references existing evidence for individualized targets, it could better contextualize how this study adds novel insights, particularly regarding PROs and patient experience. The discussion of the complex relationship between psychological and physical health is valuable but somewhat broad and would benefit from clearer linkage to the study’s specific findings. Overall, the conclusion is optimistic but would gain from a more cautious tone that clearly differentiates preliminary feasibility results from conclusive evidence.

**********

what does this mean? ). If published, this will include your full peer review and any attached files.

If you choose “no”, your identity will remain anonymous but your review may still be made public.

**Do you want your identity to be public for this peer review?** For information about this choice, including consent withdrawal, please see our Privacy Policy

Reviewer #1: No

Reviewer #2: No

**********

[NOTE: If reviewer comments were submitted as an attachment file, they will be attached to this email and accessible via the submission site. Please log into your account, locate the manuscript record, and check for the action link "View Attachments". If this link does not appear, there are no attachment files.]

While revising your submission, please upload your figure files to the Preflight Analysis and Conversion Engine (PACE) digital diagnostic tool, https://pacev2.apexcovantage.com/ . PACE helps ensure that figures meet PLOS requirements. To use PACE, you must first register as a user. Registration is free. Then, login and navigate to the UPLOAD tab, where you will find detailed instructions on how to use the tool. If you encounter any issues or have any questions when using PACE, please email PLOS at figures@plos.org . Please note that Supporting Information files do not need this step.

# Author comment: Author response to Decision Letter 2

**DOI:** 10.1371/journal.pone.0317162.r004

28 Nov 2025

14/11/25

Re: PLOS ONE Decision: Revision required [PONE-D-24-59147] - [EMID:7fa3d61f3a31db3b]

Dear Editor and Reviewers,

On behalf of my co-authors, I would like to thank you for reviewing our article entitled “Feasibility of glycated haemoglobin target-setting in adults with diabetes: a mixed-methods study” with opportunity to revise and resubmit.

We found the reviewer’s comments helpful in revising the manuscript and have carefully considered their suggestions. We revised the manuscript in accordance with the reviewer’s comments and the journal requirements.

Below, we have outlined journal requirements and reviewer comments in black followed by our responses in red, prefaced “Author response”. Corresponding changes are highlighted in the tracked manuscript text file in red using the ‘track changes’ function.

Yours Sincerely,

Dr Sam Westall

On Behalf of all the authors

Reviewers' comments and author responses (>>>):

1. If the authors have adequately addressed your comments raised in a previous round of review and you feel that this manuscript is now acceptable for publication, you may indicate that here to bypass the "Comments to the Author" section, enter your conflict of interest statement in the "Confidential to Editor" section, and submit your "Accept" recommendation.

Reviewer #1: All comments have been addressed

Reviewer #2: All comments have been addressed________________________________________

2. Is the manuscript technically sound, and do the data support the conclusions?

The manuscript must describe a technically sound piece of scientific research with data that supports the conclusions.

Reviewer #1: (No response)

Reviewer #2: Partly

________________________________________

3. Has the statistical analysis been performed appropriately and rigorously?

Reviewer #1: (No response)

Reviewer #2: Yes

________________________________________

4. Have the authors made all data underlying the findings in their manuscript fully available?

The capital PLOS data policy requires authors to make all data underlying the findings described in their manuscript fully available without restriction, with rare exception (please refer to the data availability statement in the manuscript PDF file). The data should be provided as part of the manuscript or its supporting information, or deposited to a public repository. For example, in addition to summary statistics, the data points behind means, medians and variance measures should be available. If there are restrictions on publicly sharing data- e.g. participant privacy or use of data from a third party- those must be specified.

Reviewer #1: (no response)

Reviewer #2: Yes

5. Is the manuscript presented in an intelligible fashion and written in standard English?

PLOS ONE does not copyedit accepted manuscripts, so the language in submitted articles must be clear, correct, and unambiguous. Any typographical or grammatical errors should be corrected at revision, so please note any specific errors here.

Reviewer #1: (No Response)

Reviewer #2: Yes

________________________________________

5. Review Comments to the Author

Please use the space provided to explain your answers to the questions above. You may also include additional comments for the author, including concerns about dual publication, research ethics, or publication ethics. (Please upload your review as an attachment if it exceeds 20,000 characters)

Reviewer #1: (No Response)

Reviewer #2: I am writing to inform you that I do not recall whether I have previously evaluated this manuscript or if I have been asked to provide a third opinion. Nevertheless, I have carefully read through the manuscript and have provided my comments for your consideration.

Title: The title is long and dense, making it harder to quickly digest.

>>>Title amended.

Abstract: After reading the abstract, I found it somewhat lengthy and noticed that it lacks key methodological details, particularly regarding the analysis of both qualitative and quantitative data.

>>>Abstract shortened and key methodological details included.

Background: The background effectively covers a wide range of relevant topics, including the clinical importance of glycated haemoglobin (HbA1c), the rationale for individualized targets, associated comorbidities, and healthcare system challenges. However, the section is too lengthy and somewhat repetitive, particularly in explaining the complications associated with poor glycaemic control. For instance, the negative health outcomes are listed extensively, which could be condensed for impact. In addition, the structure could be more cohesive. It jumps between patient-level challenges, healthcare provider challenges, treatment outcomes, and statistics. Grouping similar ideas together and using clearer transitions would help the reader follow the argument more easily.

>>>Background shortened and improved for better flow.

Aim and Objectives: The aims and objectives are generally clear and well-structured, reflecting the exploratory nature of the study and its focus on both clinical and psychosocial outcomes. However, the phrasing of the aim could be clearer—terms like "relaxed or intensified glycated haemoglobin targets" may be ambiguous to some readers and would benefit from clarification. The use of Roman numerals to list sub-points under the aim is somewhat confusing and unnecessary, especially as these points are already covered in the objectives. Additionally, there is some redundancy between the aim and Objective I, both of which address feasibility aspects. Objective 2 is overly broad, combining multiple outcome variables that could be better organized into psychosocial and clinical categories. Terminology such as "acceptability" and "experiences, views and opinions" should be more precise, and the inconsistent use of past and present tense between the aim and objectives could be revised for consistency. Overall, while the content is appropriate, the section would benefit from clearer wording, streamlined structure, and greater

precision in describing the study's intent and scope.

>>>Clarity improved. Redundancy removed. Precision of language improved. Tense consistency amended.

Participants: The participants section provides a basic overview of inclusion criteria and recruitment procedures but falls short in terms of methodological depth and clarity. While it is appropriate to define the target population (adults with type 1 or type 2 diabetes and specified HbA1c range), referring readers to an external protocol for exclusion criteria is not ideal; at least a summary of key exclusions should be included for transparency.

>>>Exclusion criteria now included, rather than referring the reader to the published protocol.

Recruitment: The recruitment strategy—via mailed invitations prior to clinic visits—is briefly described, but lacks Detail on how many participatory patients were approached, how many agreed to participate, and whether there were any notable patterns in non-response or attrition. The mention of face and content validity review by a service user group is a strength, yet the process and its impact on document revisions are not elaborated. Furthermore, the sampling approach for the qualitative component is only superficially described.

>>>This detail (number screened, eligible, enrolled, excluded, etc) is included in the Figure 1 CONSORT flow chart, now referenced in this section.

>>>Service user group feedback and process details included.

>>>Further detail provided on the qualitative component sampling approach.

Data Analysis: The data analysis section demonstrates a generally sound approach to handling quantitative data in a feasibility study, with appropriate attention given to descriptive statistics, data normality, and the use of inferential tests to explore trends. However, several issues limit its clarity and methodological robustness. Firstly, repeatedly referring readers to a previously published protocol diminishes the transparency of this standalone report; Key components- especially analytic decisions- should be more fully explained within the current manuscript. While the use of both parametric (t-test) and non-parametric (Wilcoxon, Mann-Whitney U) tests is appropriate based on distribution, the rationale for using inferential statistics in a feasibility study- despite acknowledging underpowered sample size- remains questionable and should be so it could be misleading if interpreted as evidence of effectiveness. More emphasis should be placed on interpreting these results cautiously. The qualitative data analysis section reflects a generally rigorous and methodologically sound approach, particularly for a feasibility study aiming to explore patient and professional perspectives. The use of the framework method of thematic analysis is appropriate for studies that involve multiple researchers and predefined areas of interest, and the integration of Nvivo software adds a layer of transparency and auditability. The strategy of combining deductive (predetermined themes) with inductive (emergent themes) coding demonstrates a balanced approach that allows for both structured inquiry and responsiveness to participant voices.

>>>Additional detail has been added instead of referring the reader to the protocol.

>>>Additional information provided on analytical processes and decisions.

>>>Further emphasis on the importance of interpreting results with caution added.

Results: The results section is clear, transparent, and responsive to reviewer concerns, with appropriate statistical analysis and a balanced presentation of findings. However, the small sample size, high dropout rate, and lack and some lack of detail (especially in qualitative reporting and integration) limit the strength and generalizability of the conclusions. For feasibility study, these are not fatal flaws, but they do highlight important considerations for future research.

>>>Additional detail provided to qualitative results. Commentary on data integration inserted in discussion, including how method triangulation, etc, enhanced credibility of findings.

Discussion: This discussion is rich in data interpretation and insight and demonstrates good scholarly rigor. To enhance clarity and impact, consider restructuring for flow, removing redundancies, expanding on key unexpected findings, explicitly linking qualitative and quantitative data, and adding a limitations section. These refinements strengthen the narrative and make the implications more accessible for readers and future researchers.

>>>Discussion shortened, restructures, and now demonstrate greater caution with result interpretation. Explicit linkages to quantitative and qualitative findings elaborated upon.

Strength and limitations: The study's strengths include a clear focus on feasibility with transparent acknowledgment of its limited statistical power, alongside thorough demographic reporting that allows assessment of generalizability within a defined local context. The use of a mixed-methods design and multiple forms of triangulation enhances the credibility and trustworthiness of the findings, demonstrating methodological rigor. However, significant limitations arise from recruitment at a single centre with a predominantly white population and higher local diabetes prevalence, which limits broader applicability of the results. The small sample size and wide confidence intervals reflect the underpowered nature of the study, restricting meaningful statistical conclusions. Additionally, attrition bias is a concern, particularly due to the higher dropout rates among younger participants whose reasons for withdrawal were often unreported. These factors weaken the internal validity and highlight the need for multicentre recruitment and retention strategies in future research to improve representativeness and reduce bias.

>>>Agreed and all discussed within strengths and limitations

Conclusion: The conclusion effectively highlights the study's contribution to the limited evidence on psychometric outcomes in diabetes and the potential benefits of individualized glycated haemoglobin targets. It appropriately emphasizes the feasibility of a larger, more definitive trial and reinforces the importance of shared decision-making In clinical practice. However, the conclusion tends to overstate the preliminary findings by suggesting improvements in patient-reported outcomes (PROs) and biomedical measures without sufficiently acknowledging the study's limited statistical power and potential biases discussed earlier. While it references existing evidence for individualized targets, it could better contextualize how this study adds novel insights, particularly regarding PROs and patient experience. The discussion of the complex relationship between psychological and physical health is valuable but somewhat broad and would benefit from clearer linkage to the study's specific findings. Overall, the conclusion is optimistic but would gain from a more cautious tone that clearly differentiates preliminary feasibility results from conclusive evidence.

>>>Flow improved. Conclusion has been adapted to adopt a more cautious interpretation of findings, acknowledging the limitations of the study. Improved contextualisation of the novel insights provided by the study.

________________________________________

6. PLOS authors have the option to publish the peer review history of their article (what does this mean?). If published, this will include your full peer review and any attached files.

If you choose “no”, your identity will remain anonymous but your review may still be made public.

Do you want your identity to be public for this peer review? For information about this choice, including consent withdrawal, please see our Privacy Policy.

Reviewer #1: No

Reviewer #2: No

------END OF RESPONSE------

Attachment Submitted filename: Response to reviewers 1125.docx

# Peer review: Decision Letter 2

**DOI:** 10.1371/journal.pone.0317162.r005

**Authors:** Yee Gary Ang

8 Dec 2025

Feasibility of glycated haemoglobin target-setting in adults with diabetes: a mixed-methods study

PONE-D-24-59147R2

Dear Dr. Westall,

We’re pleased to inform you that your manuscript has been judged scientifically suitable for publication and will be formally accepted for publication once it meets all outstanding technical requirements.

Within one week, you’ll receive an e-mail detailing the required amendments. When these have been addressed, you’ll receive a formal acceptance letter and your manuscript will be scheduled for publication.

An invoice will be generated when your article is formally accepted. Please note, if your institution has a publishing partnership with PLOS and your article meets the relevant criteria, all or part of your publication costs will be covered. Please make sure your user information is up-to-date by logging into Editorial Manager at Editorial Manager®  and clicking the ‘Update My Information' link at the top of the page. For questions related to billing, please contact billing support .

If your institution or institutions have a press office, please notify them about your upcoming paper to help maximize its impact. If they’ll be preparing press materials, please inform our press team as soon as possible -- no later than 48 hours after receiving the formal acceptance. Your manuscript will remain under strict press embargo until 2 pm Eastern Time on the date of publication. For more information, please contact onepress@plos.org.

Kind regards,

Yee Gary Ang, MBBS MPH

Academic Editor

PLOS ONE

Additional Editor Comments (optional):

Reviewers' comments:

Reviewer's Responses to Questions

**Comments to the Author**

Reviewer #1: (No Response)

Reviewer #2: All comments have been addressed

**********

2. Is the manuscript technically sound, and do the data support the conclusions?

Reviewer #1: (No Response)

Reviewer #2: Yes

**********

3. Has the statistical analysis been performed appropriately and rigorously?

Reviewer #1: (No Response)

Reviewer #2: Yes

**********

4. Have the authors made all data underlying the findings in their manuscript fully available?

The PLOS Data policy

Reviewer #1: (No Response)

Reviewer #2: Yes

**********

5. Is the manuscript presented in an intelligible fashion and written in standard English?

Reviewer #1: (No Response)

Reviewer #2: Yes

**********

Reviewer #1: (No Response)

Reviewer #2: The authors responded accurately, comprehensively, and respectfully to your feedback. They clearly show that they revised the manuscript in line with each comment, including improving methodological transparency, streamlining sections, enhancing clarity, and adopting a more cautious interpretation of findings.

**********

what does this mean? ). If published, this will include your full peer review and any attached files.

If you choose “no”, your identity will remain anonymous but your review may still be made public.

**Do you want your identity to be public for this peer review?** For information about this choice, including consent withdrawal, please see our Privacy Policy

Reviewer #1: No

Reviewer #2: **Yes: ** Chantira Chiaranai

**********

# Editor report: Acceptance letter

**DOI:** 10.1371/journal.pone.0317162.r006

**Authors:** Yee Gary Ang

PONE-D-24-59147R2

PLOS One

Dear Dr. Westall,

I'm pleased to inform you that your manuscript has been deemed suitable for publication in PLOS One. Congratulations! Your manuscript is now being handed over to our production team.

At this stage, our production department will prepare your paper for publication. This includes ensuring the following:

* All references, tables, and figures are properly cited

* All relevant supporting information is included in the manuscript submission,

* There are no issues that prevent the paper from being properly typeset

You will receive further instructions from the production team, including instructions on how to review your proof when it is ready. Please keep in mind that we are working through a large volume of accepted articles, so please give us a few days to review your paper and let you know the next and final steps.

Lastly, if your institution or institutions have a press office, please let them know about your upcoming paper now to help maximize its impact. If they'll be preparing press materials, please inform our press team within the next 48 hours. Your manuscript will remain under strict press embargo until 2 pm Eastern Time on the date of publication. For more information, please contact onepress@plos.org.

You will receive an invoice from PLOS for your publication fee after your manuscript has reached the completed accept phase. If you receive an email requesting payment before acceptance or for any other service, this may be a phishing scheme. Learn how to identify phishing emails and protect your accounts at https://explore.plos.org/phishing.

If we can help with anything else, please email us at customercare@plos.org.

Thank you for submitting your work to PLOS ONE and supporting open access.

Kind regards,

PLOS ONE Editorial Office Staff

on behalf of

Dr. Yee Gary Ang

Academic Editor

PLOS One
